# Response of Pedunculate Oak (*Quercus robur* L.) to Adverse Environmental Conditions in Genetic and Dendrochronological Studies

**DOI:** 10.3390/plants14010109

**Published:** 2025-01-02

**Authors:** Konstantin V. Krutovsky, Anna A. Popova, Igor A. Yakovlev, Yulai A. Yanbaev, Sergey M. Matveev

**Affiliations:** 1 Department of Forest Genetics and Forest Tree Breeding, Georg-August University of Göttingen, 37077 Göttingen, Germany; 2 Center for Integrated Breeding Research, Georg-August University of Göttingen, 37075 Göttingen, Germany; 3 Laboratory of Forest Genomics, Genome Research and Education Center, Institute of Fundamental Biology and Biotechnology, Siberian Federal University, 660041 Krasnoyarsk, Russia; 4 Department of Genomics and Bioinformatics, Institute of Fundamental Biology and Biotechnology, Siberian Federal University, 660041 Krasnoyarsk, Russia; 5 Laboratory of Population Genetics, N. I. Vavilov Institute of General Genetics, Russian Academy of Sciences, 119333 Moscow, Russia; 6 Scientific and Methodological Center, G. F. Morozov Voronezh State University of Forestry and Technologies, 394087 Voronezh, Russia; 7 Department of Forest Genetics, Biotechnology and Plant Physiology, G.F. Morozov Voronezh State University of Forestry and Technologies, 394087 Voronezh, Russia; logachevaaa@rambler.ru; 8 Division of Biotechnology and Plant Health, Norwegian Institute of Bioeconomy Research, NO-1431 Ås, Norway; igor.yakovlev@nibio.no; 9 Department of Forestry and Landscape Design, Bashkir State Agrarian University, 450001 Ufa, Russia; yanbaev_ua@mail.ru; 10 Ufa Institute of Biology, Ufa Federal Research Center, Russian Academy of Sciences, 450054 Ufa, Russia; 11 Department of Silviculture, Forest Inventory and Forest Management, G.F. Morozov Voronezh State University of Forestry and Technologies, 394087 Voronezh, Russia; lisovod@bk.ru

**Keywords:** adaptation, climate, dendrochronology, dendrogenomics, environment, epigenetics, genetics, genomics, plasticity, *Quercus robur*, resilience, pedunculate oak, stress

## Abstract

Pedunculate oak (*Quercus robur* L.) is widely distributed across Europe and serves critical ecological, economic, and recreational functions. Investigating its responses to stressors such as drought, extreme temperatures, pests, and pathogens provides valuable insights into its capacity to adapt to climate change. Genetic and dendrochronological studies offer complementary perspectives on this adaptability. Tree-ring analysis (dendrochronology) reveals how *Q. robur* has historically responded to environmental stressors, linking growth patterns to specific conditions such as drought or temperature extremes. By examining tree-ring width, density, and dynamics, researchers can identify periods of growth suppression or enhancement and predict forest responses to future climatic events. Genetic studies further complement this by uncovering adaptive genetic diversity and inheritance patterns. Identifying genetic markers associated with stress tolerance enables forest managers to prioritize the conservation of populations with higher adaptive potential. These insights can guide reforestation efforts and support the development of climate-resilient oak populations. By integrating genetic and dendrochronological data, researchers gain a holistic understanding of *Q. robur*’s mechanisms of resilience. This knowledge is vital for adaptive forest management and sustainable planning in the face of environmental challenges, ultimately helping to ensure the long-term viability of oak populations and their ecosystems. The topics covered in this review are very broad. We tried to include the most relevant, important, and significant studies, but focused mainly on the relatively recent Eastern European studies because they include the most of the species’ area. However, although more than 270 published works have been cited in this review, we have, of course, missed some published studies. We apologize in advance to authors of those relevant works that have not been cited.

## 1. Introduction

The genus *Quercus* includes about 450 species of both deciduous and evergreen forest trees, the majority of which are distributed mainly from northern latitudes to the tropics [1]. The pedunculate oak (*Quercus robur* L.), also known as the English or European oak, occupies a large area in Europe [2] and plays a significant role both ecologically and economically [3]. It is a keystone species that provides vital habitat and resources for many other organisms from insects and birds to mammals and fungi. Over 2300 species have been associated with oaks, including specific insects, such as moths, butterflies, and beetles, that rely on oak leaves, acorns, or bark [4]. These trees are excellent habitats, particularly for woodland birds, bats, wild boar, and small mammals. The acorns provide food for animals like squirrels, jays, and deer, while older trees with hollow trunks create nesting and roosting sites for bats and birds. As oak leaves decompose, they enrich the soil with organic matter, benefiting the entire forest floor ecosystem. Oak leaf litter also supports many detritivores (decomposer organisms) that break down dead organic material, which in turn supports soil health and fertility.

Oak trees store substantial amounts of carbon in their wood, leaves, and roots, helping reduce greenhouse gases in the atmosphere and mitigating global climate warming [5,6,7,8]. Their large canopies also help in regulating local microclimates by providing shade and retaining moisture in the surrounding area [9,10,11]. Oaks also play an important role in water regulation by absorbing rainfall, reducing runoff, and maintaining soil structure, which helps prevent erosion [12,13].

Oak wood is highly valued for its durability, strength, and attractive grain [2,14,15]. *Q. robur* wood is used in furniture, flooring, barrels (for wine and whiskey aging), and high-quality construction materials [2,16,17,18,19,20]. Its resistance to moisture and decay also makes it suitable for outdoor use, including fencing and shipbuilding [2,21].

Large, ancient oaks are often landmarks and attract tourists for recreational activities like hiking, birdwatching, and photography [22,23]. This contributes to local economies, especially in areas known for scenic oak woodlands.

Although less common today, oak wood has historically been used as firewood and charcoal due to its high energy content and slow-burning nature [24,25,26]. In some regions, it still remains a preferred wood for sustainable fuel. The bark and leaves of the oak have been traditionally used for their tannins, which have medicinal applications in treating skin issues and inflammation [27,28,29]. Additionally, the oak has cultural significance in many European countries, symbolizing strength, endurance, and longevity [30,31,32,33].

Overall, the *Q. robur*’s ecological contributions help sustain biodiversity and ecosystem balance, while its economic value supports industries from timber production to tourism. However, the *Q. robur* forests are threatened and declining in many areas due to a combination of bad weather conditions, phytopathogens, pest outbreaks, and repeated severe defoliation likely associated with climate change [34,35,36,37,38,39,40,41,42,43,44,45]. That is why it is extremely important to study response of *Q. robur* to adverse environmental and weather conditions and to search for its adaptive potential using genetic, genomic, and dendrochronological methods and, especially, interdisciplinary approaches, such as dendrogenomics [46]. Available genomic, transcriptomic, and experimental resources for oaks, including *Q. robur*, and applications of these resources to genetic linkage and Quantitative Trait Locus (QTL) mapping and to population genomic analyses, such as association mapping (Genome-wide Association Analyses, GWAS), landscape genetics, population epigenomics, paleogenomics, and phylogenomics studies, are reviewed also in [47]. This review emphasizes the application of genomic tools for exploring local adaptation, adaptive divergence, and interspecific hybridization and introgression in oaks. It particularly focuses on recent population genomics approaches that address the genomic basis of adaptive trait variation and local adaptation. Additionally, it examines the roles of interspecific hybridization and epigenetic changes in facilitating rapid local adaptation, lineage divergence, and speciation, alongside the temporal scale of adaptive genomic shifts. Prospective advancements in genome-wide variation studies, such as whole-genome resequencing at the population level, high-resolution population epigenomics, genomic vulnerability assessments, and strategies for conservation and sustainable management of oak populations amid environmental changes, are briefly discussed [47].

Herein, we present a summary on basic biological, ecological and genetic features of *Q. robur*, but focusing on its response to adverse environmental and weather conditions in genetic and dendrochronological studies. One of the main objectives of the review is to summarize also publications on *Q. robur* genetic diversity and adaptive traits that can guide reforestation efforts and help develop climate-resilient oak populations. The review is focused on genetic and dendrochronological studies that offer complementary perspectives on tree response mechanisms. Genetic studies reveal adaptive genetic diversity and inheritance patterns, while dendrochronology provides a phenotypic record of responses over time. Combining these fields creates a holistic understanding of how *Q. robur* responds to stress, useful for fields ranging from ecology to forest genetics. Hopefully, this review helps understand *Q. robur*’s resilience mechanisms, support adaptive management practices, and enable sustainable forest planning in the face of environmental challenges.

## 2. Nuclear Genome of *Q. robur*

*Q. robur* has a diploid set of 24 chromosomes (2n = 2x = 24) [48]. Triploids among *Q. robur* trees were found only in a few cases [49,50,51]. An additional B chromosome was observed in some studies (2n = 2x = 24 + 1B) [52,53]. Analyses of the karyotypes of the genera *Quercus*, *Lithocarpus*, *Castanopsis* and *Castanea* [54,55], and *Fagus* [56,57] by different research groups showed that the number of chromosomes in the Fagaceae family is generally stable and amounts to 2n = 24. The oak karyotype contains two types of chromosomes: seven pairs of metacentric and five pairs of submetacentric [58]. Current oak chromosomes evolved through multiple fusions, fissions, and rearrangements after an ancestral triplication event [59]. No secondary constriction or satellites were detected, but regular association of the nucleolus with one of the chromosome pairs was detected in meiotic observations. Based on this, it can be assumed that the nucleolus-forming regions are located in the telomeric regions of this chromosome [60].

The DNA content in different species of the genus *Quercus* varies per 1C from 0.5 pg (490 Mbp) in *Q. sessilis* to 1.22 pg (1195 Mbp) in *Q. austrochinchinensis* with a mean of 0.95 (±0.13) pg (924 ± 131 Mbp) for 29 species according to [61]. The DNA content of *Q. robur* per 1C was 0.90 pg (882 Mbp) [62]. According to [63], the DNA content among 28 species of the genus *Quercus* varies per 1C from 0.59 pg (565 Mbp) in *Q. velutina* to 1 pg (980 Mbp) in *Q. coccifera* and *Q. suber*. The studied species include representatives of all four main subgenera or sections: 11 species of the subgenus *Erythrobalanus* (red oak), 12 species of the subgenus *Lepidobalanus* (white oak), three species of the subgenus *Cerris*, and two-subgenus *Sclerophyllodrys*. The average DNA content was 0.79 pg (759 Mbp) for the subgenus *Erythrobalanus*, 0.85 pg (0.785 Mbp) for *Lepidobalanus*, and 0.87 pg for *Cerris*. Both species *Q. coccifera* and *Q. ilex* of the subgenus *Sclerophyllodrys* had a maximum genome size of 1 pg (980 Mbp). They are evergreen plants and belong to the botanical group of the subgenus *Sclerophyllodrys* according to the classification [64]. But according to another classification [65], they belong to two different subgenera: *Q. ilex* belongs to the subgenus *Lepidobablanus*, and *Q. coccifera* to the subgenus *Cerris*. Molecular genetic analysis also included these two evergreen oak species in different subgenera [66,67], confirming the previously made taxonomic division of the *Sclerophyllodrys* group [64].

The earlier studies on the genetic linkage mapping of *Q. robur* are described in [68,69,70,71,72,73] and summarized in [74]. The densest genetic linkage map for the genus *Quercus* is based on 4261 single-nucleotide polymorphism (SNP) markers obtained by genotyping more than 1000 full-sibs from two intraspecific (*Q. robur*: 3PxA4, *Q. petraea*: QS28xQS21) and two interspecific full-sib families of *Q. petraea* and *Q. robur* (11PxQS29 and 11PxQS28) by using an 8K gene-based SNP array [75]. A single composite map was constructed by merging the eight parental maps using LPMerge software [76]. This is spanning 742 cM over the 12 linkage groups (LGs) of the oak genome. Overall, 82% of the SNPs successfully genotyped were polymorphic in at least one of the four pedigrees, and 63% were mapped as framework markers on the parental genetic maps [44]. This oak map establishes a foundation for genome-wide analysis at the centimorgan level, supporting studies on evolutionary relationships among related species, genomic scans of species and population divergence, and the positional identification of quantitative trait loci (QTLs) through co-localization with gene-based markers. It also aids in identifying chromosomal rearrangements [73].

The development of the next-generation sequencing (NGS) techniques greatly promoted the whole genome sequencing, including oaks. First complete assembly and annotation of the *Q. robur* genome consisted in a total of 17,910 scaffolds (>2 Kb each) corresponding to ~1.354 Gbp/2C [77]; the European Nucleotide Archive accession number ERP006803; available also online at https://www.oakgenome.fr/index4f22.html?page_id=244, accessed on 14 October 2024). The second version contained 8827 scaffolds covering 1.455 Gbp [78] and 871 scaffolds assigned to the 12 linkage groups of the genetic map covering 716.6 Mb and containing 23,220 genes [79]). The current genome assembles of *Q. robur* available at the NCBI GenBank database are presented in Table 1.

## 3. Chloroplast Genome of *Q. robur*

The chloroplast genome of the *Q. robur* has been studied extensively due to its significance in understanding the evolutionary history, genetic diversity, and adaptation of this species across Europe. The chloroplast genome of *Q. robur* is typical of angiosperms, featuring a circular structure with approximately 160,000 base pairs. It contains the standard set of genes necessary for photosynthesis and other chloroplast functions, along with inverted repeat regions that help maintain genomic stability [80].

Research on the chloroplast genome has revealed geographic structure, with different haplotypes fixed in regional populations [81]. This structure provides insight into how *Q. robur* recolonized Europe following the last Ice Age [82]. Chloroplast DNA variation in *Q. robur* shows a clear east-west gradient, suggesting multiple refugial sources and migration pathways [81,83,84,85,86,87]. This geographic pattern also helps explain the resilience and adaptability of the species to various climates and habitats in Europe.

The chloroplast genome’s high differentiation among populations contrasts with the nuclear genome, which tends to show more within-population diversity [82]. This differentiation has important implications for conservation, as it emphasizes the need for preserving regional genetic diversity to maintain resilience in *Q. robur* populations. Conservation strategies often recommend using local genetic material for reforestation and avoiding the introduction of non-local genotypes, which could disrupt local adaptations.

Chloroplast DNA markers from *Q. robur* have been used in population genetics to understand interspecies hybridization, notably with *Q. petraea*. These markers are essential for distinguishing species and assessing the genetic purity of populations, which is crucial in forestry and conservation management [88].

The chloroplast genome of *Q. robur* is a crucial aspect of its genetic makeup, providing insights into the species’ adaptation, evolution, and phylogeography [81,82,83,84,85,86,87,88,89,90]. This chloroplast genome, transmitted maternally, helps trace lineage and historical distribution across Europe, particularly as this oak species migrated northward after the last Ice Age [88]. Chloroplast DNA variation in *Q. robur* shows geographic structuring, with certain chloroplast haplotypes becoming fixed in specific populations due to limited gene flow through seeds and high regional differentiation in chloroplast genomes compared to nuclear genomes [84,85,86,87,88,89,90]. This structure has contributed to the east-west genetic gradient observed in the species and is important for understanding historical migration routes and the oak’s adaptability to different environments.

One significant application of understanding chloroplast DNA in *Q. robur* is its role in conservation genetics. As climate changes and habitat fragmentation continue, the genomic diversity within chloroplasts supports resilience and adaptation, guiding conservation efforts. Effective management practices, such as promoting local seed use for regeneration, help maintain this diversity and prevent the introduction of non-native genotypes, which could disrupt local adaptations in *Q. robur* populations.

Additionally, the high level of phenotypic variation tied to the chloroplast genome helps *Q. robur* thrive in diverse environments, from rocky slopes to sand dunes and peat bogs. This adaptability is partly due to chloroplast variations supporting efficient photosynthesis and other cellular functions, which are essential for survival in different ecological niches.

The genetic insights gained from the chloroplast genome highlight the importance of preserving genetic diversity within oak populations, particularly as threats from pathogens, pests, and climate pressures increase. These findings suggest a continued focus on genetic conservation and sustainable management practices to ensure the resilience of oak forests in Europe.

In summary, the chloroplast genome of *Q. robur* plays a vital role in tracing historical migration, supporting biodiversity conservation, and providing insights into the evolutionary resilience of this ecologically important tree. For more detailed information, see studies on chloroplast DNA variation and phylogeography in *Q. robur* by [84,85,86,87,88,89,90].

## 4. Mitochondrial Genome of *Q. robur*

The mitochondrial genome of oak species is supposedly comparatively complex, particularly due to the large size of plant mitochondrial genomes and the presence of numerous non-coding regions. Currently, the complete mitochondrial genome assemblies are only for four oak species: *Q. robur* of 390,906 bp (the NCBI GenBank accession number OW028777.1), Chinese cork oak (*Q. variabilis*) of 412,886 bp [91] (the NCBI GenBank accession number MN199236.1) and of 419,744 bp [92] (the NCBI GenBank accession number CP129458.1), Japanese sawtooth oak (*Q. acutissima*) of 448,982 bp [93] (the NCBI GenBank accession number MZ636519.1), and cork oak (*Q. suber*) of 531,858 bp [94]. The mitogenome of *Q. acutissima* is presented by three distinguished contigs including a single linear molecule and two circular molecules with 448,982 bp total length [93]. It contains 51 genes, including 32 protein-coding, 16 tRNA, and three rRNA genes. The complete mitochondrial genome of *Q. variabilis* is supposedly circular and contains slightly more genes—60 in total, including 36 protein-coding, 21 tRNA, and three rRNA genes [91]. The mitogenome of *Q. suber* is presented by three distinguished linear contigs including one large contig with 442,094 bp and two smaller contigs of 52,064 bp and 37,700 bp, respectively [94]. It contains a total of 66 genes, including 40 protein-coding, 23 tRNA, and three rRNA. The mitogenome of *Q. robur* is the shortest and presented by a single linear contig, but it is not verified and annotated yet. However, studies indicate that the mitochondrial genome of *Q. robur* might have considerable variation in its structural organization, contributing to genetic diversity across oak populations. Both genomes chloroplast and mitochondrial are maternally inherited in *Q. robur* [80].

Studies of the mitochondrial markers help understand evolutionary relationships, population differentiation, and adaptation strategies in plant species [95]. Studies of *Q. robur* populations based on the mitochondrial markers are insufficient, while they can provide valuable population genetic data. However, because oak mitochondrial genomes undergo complex rearrangements and are highly repetitive, further efforts on sequencing and assembly of the *Q. robur* mitogenome are necessary for detailed insights into its genetic and evolutionary roles. This mitochondrial genome complexity is not unique to oaks; many tree species exhibit similarly large and complex mitochondrial genomes, which challenge sequencing and interpretation but are crucial for insights into plant genetics and adaptation strategies [96,97,98,99,100].

## 5. Genetic Variation and Population Structure of *Q. robur*

The genetic variation and population structure of *Q. robur* have been extensively studied due to its importance in forest ecosystems and its wide distribution across Europe. Studies highlights significant genetic diversity within populations, with studies showing high intra-population diversity (up to 98.5% of the total genetic variability), suggesting a robust capacity for adaptation within local populations [101,102,103,104]. For instance, analysis of allozyme polymorphism in isolated populations on the eastern border of *Q. robur* showed that of the total variability of the two populations, 94.6% was represented by the intrapopulation component, while interpopulation variability was only 3.4% [101]. Later, it was confirmed by SNP markers: analysis of 327 nuclear SNPs in 97 populations on the eastern part of *Q. robur* distribution range showed that of the total variability, 92.8% was represented by the intrapopulation component, while interpopulation variability was only 7.2% [104]. The total mean value of observed heterozygosity for the 12 allozyme loci was 0.229 and 0.161 in the *Q. robur* populations in Central and Eastern Europe, respectively [105]. In Croatia, a study on peripheral populations revealed that these oaks have high levels of nuclear and chloroplast genetic variation, with 66 unique chloroplast haplotypes detected across populations, a likely result of historical recolonization events and gene flow among populations. However, despite this diversity, inter-population differentiation remains relatively low, attributed to ongoing gene flow and hybridization with *Q. petraea*, which further enriches genetic diversity within mixed oak forests [106].

Analysis of genetic polymorphism of oak populations growing in Russia using random amplified polymorphic DNA (RAPD) markers revealed that the total genetic variation varied from 0.202 in Voronezh populations to 0.245 in Great (Velikij) Novgorod, which corresponded to the estimates for populations of this species from Central and Western Europe [107]. These populations showed low interpopulation differentiation (*G_ST_* = 0.098) and relatively high gene flow (*N_e_m* = 4.61). The proportion of among-population variation accounted for 7% of the total variation; more than 93% of the total variation was explained by individual and intrapopulation variation [107]. Similarly, the ISSR (Inter Simple Sequence Repeat) markers and allozyme loci showed that the lowest polymorphism was found for the Trans-Ural oak groves [108]. High polymorphism of populations was shown for geographically marginal stands, comparable to or exceeding the diversity of ISSR fragments in the oak grove in the Ufa forestry (65.6%). For some populations, maximum DNA polymorphism was identified, exceeding 80% [108]. Studies of different biotope affiliation of stands from ravine and floodplain oak groves in the south of Russia based on the analysis of the structure of five ISSR primers showed that their genetic differences were statistically significant, in particular, heterozygosity was lower in ravine oak groves [109].

The genetic study of several populations and groups of *Q. robur* in Bosnia and Herzegovina using four nuclear microsatellite markers confirmed a higher heterozygosity in these populations compared to the populations of *Q. robur* in Western Europe [110]. The authors explained it by the proximity of the studied populations to their glacial refuges. This may be a reason for their higher resistance and endurance compared to the populations of Western and Central Europe.

Despite the strong reduction and fragmentation of *Q. robur* forests over the last several centuries and comparatively small sizes of present stands in Eastern Europe, a high within population variation but relatively low differentiation between populations have been also observed in the seven *Q. robur* populations located in the basins of the Volga River and its two main tributaries, Kama and Belaya, based on the nuclear SNPs genotyped using double digest restriction site-associated DNA sequencing (ddRADseq) [111]. The large number and age diversity of local populations, as well as the complexity of migration routes of common oak from Pleistocene refugia and secondary Holocene shelters, may be the cause of this phenomenon. However, population differentiation and heterogeneity were statistically significant and should be taken into account in seed zoning and reforestation.

The study of 17 Croatian *Q. robur* populations representing southern range peripheral populations using 10 nuclear and nine chloroplast SSRs also found high within population variation for both sets of markers, and relatively low between population differentiation for nuclear, but high for chloroplast markers [102]. Isolation by distance explained 19.6% of haplotypic chloroplast differentiation. Analysis of within and between lineages showed that original recolonization patterns of nuclear diversity were subsequently erased by gene flow.

Meanwhile, the population structure and differentiation of *Q. robur* is clearly observed at the large scale [103,104,110]. A clear spatial trend was observed in the estimates of genetic diversity and differentiation of Italian *Q. robur* populations [112,113].

*Q. robur* populations are also affected by isolation-by-distance effects, contributing to some genetic differentiation among populations. For instance, studies show that approximately 19.6% of genetic differentiation can be explained by geographic distance between populations [68]. Additionally, genetic markers, such as simple sequence repeats (SSRs), have been used to assess genetic structure, revealing variations in allelic diversity that reflect both local adaptations and historical dispersal patterns [114]. Understanding these patterns is essential for conservation, especially as peripheral populations face heightened selection pressures from climate change, which could affect their genetic resilience and adaptive potential.

The effect of past environmental changes on the demography and genetic diversity of natural populations was investigated across seven widely distributed and ecologically contrasting phylogenetically distant European tree species, including *Q. petraea*, using comparative population genomic analyses and demographic inferences based on concerted sampling of 164 populations across their natural ranges [115]. For all seven species studied, the effective population size (*N_e_*) either grew or remained constant across numerous glacial cycles, extending as far back as 15 million years in the most extreme cases. The extensive genealogy of *Q. petraea* and its relatively high *N_e_* is likely due to long generation times and also results from ongoing hybridization with other abundant, closely related white oak species, including *Q. robur*. Interestingly, despite the significant environmental changes brought about by the Pleistocene glacial cycles and substantial shifts in geographic ranges, the genetic diversity of dominant forest tree species has remained largely unaffected. Altogether, these results indicate that forest trees have been able to retain their evolutionary potential over very long periods of time despite strong environmental changes.

Mining genome-wide DNA sequences enabled the discovery of a subset of 38 SNPs that are near-diagnostic markers for species assignment in four European white oaks (10 for *Q. petraea*, seven for *Q. pubescens*, nine for *Q. pyrenaica*, and 12 for *Q. robur*, respectively) despite their low interspecific differentiation [116]. These near-diagnostic markers, which are nearly species-specific, are almost completely fixed in one species and absent in the other three. They offer an efficient, reliable molecular tool for identifying *Q. petraea*, *Q. robur*, *Q. pubescens*, and *Q. pyrenaica*, making them suitable for routine use in species identification, taxonomic classification, and applications in forest research and management.

In total, 31.9 million SNPs were detected using whole-genome sequence data from pools of individuals of four species (*Q. robur*, *Q. petraea*, *Q. pyrenaica*, and *Q. pubescens*) resequenced at 400× coverage [74]. In addition, 30 million SNPs were detected using whole-genome sequence data from 18 *Q. petraea* populations resequenced at >100× coverage.

These findings underscore the importance of studying and conserving genetic diversity in *Q. robur* populations to enhance forest resilience and adaptability under environmental changes. They provide crucial insights for forest management and conservation strategies, especially for maintaining genetic resources in fragmented or climate-sensitive regions.

### Introgressive Hybridization

Early studies of introgressive hybridization between *Q. robur* and *Q. petraea* using chloroplast, mitochondrial, and nuclear (allozymes and SSRs) markers are reviewed in [88]. In a large-scale genetic study of *Q. robur* that used 1970 *Q. robur* trees from 197 locations in 13 European countries and 1763 putative *Q. petraea* trees genotyped with the same 381 polymorphic markers (356 nuclear SNPs, three nuclear InDels, 17 chloroplast SNPs, and five mitochondrial SNPs), a strong spatial pattern with a highly significant autocorrelation up to a range of 1250 km was found for both organelle and nuclear markers [106]. However, the differentiation at the nuclear gene markers was much lower compared to the organelle gene markers. Two main genetic clusters were found. The western gene pool was significantly influenced by introgression from *Q. petraea* in the past. In Germany, a contact zone for *Q. robur* with varying levels of introgression was identified, likely due to differing historical introgression levels in glacial refugia or during postglacial recolonization. The primary postglacial recolonization routes moved from south to north and south to northwest in Western and Central Europe, while eastern haplotypes spread from east to west across Central Europe. In contrast, pollen-mediated gene flow and introgression from *Q. petraea* significantly altered the large-scale nuclear gene structure, showing a pronounced west-to-east pattern. Asymmetric nature of interspecific hybridization between *Q. robur* and *Q. petraea* was observed in mixed stands in Southern Lithuania using SSR and RAPD markers [117].

Introgressive hybridization of *Q. robur* with other oak species, notable with sessile oak (*Q. petraea*) may be an important source of new alleles for adaptation in rapidly changing environments, perhaps even more important than constant variation [84]. Hybridization and adaptive introgression seem to be major drivers of ecological success of oaks [118,119,120]. Similar conclusions have been done for two closely related and introgressing spruce species (*Picea abies* and *P. sibirica*) [121,122]. It is important to note that, based on analyses of historical and recent gene flow events between *Q. petraea* and *Q. robur* using two independent methods—diffusion approximation to the joint frequency spectrum and approximate Bayesian computation (ABC)—and 3524 randomly distributed SNPs across the genome, these species evolved in isolation for most of their history. They only recently entered secondary contact, likely due to the latest phase of postglacial warming [123]. Research indicates that substantial genetic differentiation existed before this secondary contact, allowing development of barriers to gene flow. Consequently, the genomes of modern European white oaks exhibit a mosaic structure, containing both genes that have crossed species boundaries and genes resistant to gene flow.

It is interesting that for two oak species, *Q. acutissima* and *Q. variabilis*, it was determined that the introgressed genomic adaptation signatures were predominantly localized in regions with suppressed recombination rates [124]. Introgression likely promotes adaptation in these oak populations by introducing allelic variations in cis-regulatory elements, particularly through the insertion of transposable elements, thereby altering the regulation of stress-related genes. These results open new avenues for studying mechanisms of hybridization-mediated adaptation in sympatric species.

## 6. Epigenetic Variation of *Q. robur*

Oak species, including *Q. robur*, have been actively studied for the epigenetic variation both in general and as a response to environmental stimuli [125,126,127,128,129]. Epigenetic variation plays a vital role in the adaptability of oaks to diverse and changing environments over their long lifespans [125,127]. Through epigenetics mechanisms such as DNA methylation, histone modification, and small RNA regulation, oaks can adjust gene expression and fine-tune their physiological and developmental processes in response to stress without altering their genetic code. These epigenetic changes not only aid in immediate acclimatization but can also be inherited, providing future generations with a pre-existing ability to cope with environmental challenges.

DNA methylation is the most studied epigenetic process in the tree species [130]. Most early studies of DNA methylation were implemented in the Californian oaks [126,131]. Three populations of valley oak (*Q. lobata*) have been studied to assess whether methylation could be involved in response to the environment using reduced-representation bisulfite sequencing (RRBS). A significant differentiation among populations at single-methylation variants of the CpG context was found, which was much higher than differentiation determined in the underlying DNA sequence at single-nucleotide polymorphisms (SNPs) [126,131]. Methylation polymorphisms of the CpG context plays a more important role in local adaptation, while CHG context methylation looks to be less important targets for natural selection, either directly or through linkage to regions under selection [132]. These findings are consistent with the notion that the environmental conditions of different locations are responsible for different patterns of methylation and created high levels of epigenetic differentiation among these populations [126].

Epigenetic regulation of gene activity in plants is shaped by their evolutionary background, developmental strategies, and lifestyle. This initial exploration into the epigenetic chromatin landscape of the long-lived oak species *Q. robur* has revealed some unique characteristics when compared to other angiosperms, showing more similarities to long-lived gymnosperms such as *Pinus* and *Picea* [133]. *Q. robur* displays distinctive traits in both genome structure and its epigenetic landscape, setting it apart from other plant species. Ongoing studies of its genome and epigenome are anticipated to shed new light on the interactions between sequence composition, chromatin organization during interphase, epigenetic markers, and the functional aspects of this unique plant genome [134].

Characterization of chromatin in cyclin cells of *Q. robur* revealed that DNA methylation was not restricted to constitutive heterochromatin but was associated with both euchromatic and heterochromatic domains. Multiple chromatin modification showed dispersed distribution along chromatin. While H3K9ac (i.e., gene activating mark) exhibited typical euchromatin-specific distribution, the H3K9me1 and H3K27me2, both heterochromatin-associated marks (i.e., gene repressive marks), were not restricted to chromocenters, but showed additional dispersed distribution within euchromatin. It has been suggested that intermediate heterochromatin, containing both silencing and activation marks, is heritably maintained in plants, providing a particular epigenetic flexibility and contributing to epigenetic defense mechanisms [134].

Analysis of transcriptome, methylome, and small RNA data for two oak species with contrasting levels of drought tolerance (*Q. robur* and *Q. petraea*), under control and drought stress conditions, revealed high differences between species, consistent with species-specific response to drought stress [135]. After investigation of the methylation dynamics under the moderate drought conditions, in total, there was less differentially methylated regions (DMRs) in *Q. robur* with very low overlap of common DMRs between species. The highest difference was found in CHH context, considering the higher importance of CHH methylation for adaptive responses.

Similarly, small RNAs populations were different between species and the number of common sRNAs under drought stress were visibly reduced in *Q. robur*. It is considered that *Q. robur* deployed different molecular mechanisms in response to drought stress, consistent with the greater sensitivity to drought of this species. An integrative approach of the three data sets revealed genomic co-locations of potential importance for forest three adaptation to drought stress [135].

Oaks display a high degree of phenotypic plasticity, which allows them to thrive in diverse environments ranging from wetlands to dry forest stands [136,137,138,139]. This adaptability is partially driven by epigenetic changes that regulate genes involved in stress tolerance, growth regulation, and metabolic adjustments [135]. As shown above, the epigenetic marks can change in response to environmental stressors like drought or high temperatures, enabling oaks to adjust physiological processes such as stomatal regulation, water use efficiency, and leaf morphology [135].

A strategy for studying epigenetic diversity in natural populations of *Q. petraea*, as a concept oak species, is outlined in [130]. However, for oaks and other forest tree species, information on the importance of epigenetics in their response to the environment is sparse because their longevity and other life-history traits make experimental approaches difficult. Nevertheless, because of this longevity and wide ecological ranges, epigenetic processes may be as important, or even more important, than genetic processes in shaping responses to rapidly changing environments.

## 7. Transcriptome and Gene Expression of *Q. robur*

With the development of NGS and RNA sequencing, the accumulation of gene expression data has accelerated significantly. For example, Lesur et al. [140] created a complete gene expression catalog for oak based on the functional annotation of 91,000 unique transcriptome contigs (transcripts or unigenes) of *Q. robur* and *Q. petraea*. Transcriptome analysis showed that most of the transcriptome is common to all tissues, but tissue-specific transcripts were also identified [140]. Transcriptome analysis in oak bud tissues showed that gene expression patterns in dormant and swelling buds are different. In the buds entering dormancy, genes associated with resistance to cold stress and water deficit are overexpressed, while in swelling buds, genes associated with cell division and development are overexpressed [140,141]. This analysis provided important information about oak genome evolution and regulation of genes related to vegetative bud phenology, an important adaptive trait in trees. This resource contributes to the annotation of the oak genome sequence and will probably help link genotypes to adaptive traits. Annotated transcriptomes derived from different tissues under a variety of treatments are listed for 10 oak species, including *Q. robur* in Table 1 in [47]. The RNA sequencing data on roots of oak trees were integrated into a single comprehensive database, named OakRootRNADB that contains information on both coding and noncoding RNAs [142]. The sequences in the database also provide information on transcription factors, transcriptional regulators and chromatin regulators, as well as a prediction of the cellular localization of a transcript.

Studying the transcriptome and gene expression of oaks is critical for understanding their responses to climatic factors and environmental stresses. These studies help identify the genes and molecular pathways that govern traits such as drought tolerance, temperature adaptation, and resistance to pests and pathogens. By analyzing changes in gene expression under stress conditions, researchers can better understand oak stress responses and identify how oaks perceive and respond to environmental challenges at the molecular level. These studies help to uncover adaptive mechanisms and reveal the genetic basis of adaptive traits, such as enhanced water-use efficiency or tolerance to extreme temperatures, providing insights into how oaks survive in diverse and changing environments. Identifying stress-related genes and pathways aids in selecting resilient oak trees and populations for breeding, conservation, and reforestation, ensuring their long-term survival under climate change.

## 8. Phenotypic Variation and Plasticity of *Q. robur*

Tree phenotype is a product of its genotype and the interaction of genotype with the environment. The latter represent phenotypic plasticity and arises in response to changes in growing conditions and is manifested in changes in growth, shoot length, phenology shift, the ratio of male and female shoots in monoecious trees, and other reactions to the environment. *Q. robur*, a species with broad continental distribution range, demonstrated high phenotypic plasticity of the adaptive traits suggesting that this species has a great potential to adapt to environmental changes that may occur from global warming and indicates good perspectives for oak gene conservation and tree breeding [143,144,145].

When studying variability in tree species, three groups of traits are studied as the main ones: structural (morphological), reflecting the structural features of the shape and size of organs and their parts; physiological, characterizing the features of physiological and biochemical processes; biochemical and molecular genetic changes at the genome level.

Intraspecific variation of *Q. robur*, represented by numerous phenological, ecological, and morphological forms, varieties, etc., is the basis for the search, conservation, and rational use of its genetic potential. According to the type of crowns, the following biotypes are distinguished in *Q. robur* [146]: (1) spreading type (P-type)—developed crown with powerful lower branches; (2) umbrella-shaped type (Z-type)—crown without lower branches lost in the process of competition; (3) narrow-crown type (U-type)—a small crown, usually formed by underdeveloped secondary branches that replaced the primary crown.

Semerikov and Glotov [147] used numeric morphological traits of oak leaves and generative organs—fruit stalk, cupule, acorns—to characterize oak variability, which allowed them to identify six population groups in the Caucasus and five population groups of *Q. robur* in the Volga and Cis–Urals regions.

Gneusheva et al. [148] showed the informativeness of relative values of the shape and size of leaf blades of *Q. robur* to establish the intraspecific taxonomic rank of its populations and their geographical and ecological fragments.

Seeds of *Q. robur* have significant variation in morphometric parameters. Morphological forms of acorns are distinguished as large-fruited and small-fruited forms of acorns. According to their shape, they are divided as (1) cylindrical, (2) teardrop-shaped, (3) conical, (4) elliptical, oblong, (5) barrel-shaped, and (6) round [149]. However, the shape coefficient of acorns is not associated with shape diversity [150]. It is noted that acorns of saline oak groves are characterized by poor survival rate and low growth of the first-year seedlings in height. According to [151], the highest germination of *Q. robur* acorns was noted for small forms (90%), the germination percentage for medium-sized acorns was 82%, large ones—78%.

Acorn yield is characterized by significant interannual variations. *Q. robur* belongs to the species in which the number of acorns is determined not by the number of emerged flowers, but by the number of remained pollinated flowers and mature acorns, which determines the yield of the year (masting year) [152]. The study presents clear evidence of maternal choice during acorn development in *Q. robur*, demonstrating the positive effects of pollen supplementation. It also confirms that *Q. robur* exhibits masting behavior in fruit maturation; at this site, the occurrence of a mast year is determined by the abortion of pollinated flowers and immature acorns, rather than by the quantity of flowers produced. Taking into account the periodicity and variability of flowering and pollen production in the oaks, as well as the fact that not all trees of the same species in a population produce good-quality pollen, it is necessary to preserve the viability of pollen by storing it under certain conditions for targeted acorn production and breeding, since this allows to have a good yield [153]. Moreover, it is important to note that the patterns of spatial, temporal, and individual variability of *Q. robur* pollen grain viability is under the influence of climatic factors, which are showing compelling changing trends from year to year [154].

In conclusion, the study of *Q. robur* reveals its remarkable phenotypic plasticity and genetic diversity, enabling adaptation to environmental changes such as global warming. Phenotypic traits, shaped by both genetic and environmental factors, include morphological (e.g., crown types, leaf traits), physiological, and biochemical characteristics. Intraspecific variation, such as different crown forms and acorn traits, underscores its genetic potential for conservation and breeding efforts. Research on acorn morphology and germination highlights significant variability influenced by climatic and environmental conditions, with smaller acorns often showing higher germination rates. Additionally, *Q. robur* exhibits masting behavior in fruit production, driven by factors like pollination efficiency and flower abortion. Understanding the variability in flowering, pollen viability, and acorn yield, influenced by climatic trends, is essential for sustainable management and breeding strategies. This knowledge supports targeted conservation, enhancing the species’ resilience and ecological functions.

## 9. Phenology of *Q. robur*

Phenological analyses in populations of *Q. robur* (phenophase of leaf unfolding, flowering and leaf fall, duration of vegetation) revealed more significant differences between trees and populations than between years of study [153]. The same phenological form was also noted in individual trees within populations, which indicates their supposed common origin [153]. Based on this, it can be concluded that the differences between populations are due to high intraspecific variability, i.e., the phenological form of the population (phenoform) [153,154,155]. The onset of the phenophase is largely under genetic control, and this is important for the classification of source material in the process of forest tree breeding [153]. The phenomenon of polycyclism is also characteristic of oak—the ability to begin growth several times during the same growing season [156,157]. Polycyclism of *Q. robur* also includes a rare phenomenon such as summer flowering [153].

There are different varieties (forms) of *Q. robur*: “early” and “late”. The early (*Q. robur* L. f. praecox Czern.) and late (*Q. robur* L. f. *tardiflora* Czern.) phenological forms were first described by E. F. Zyablovsky in 1804 and V. M. Chernyaev in 1858 [158]. In addition to these extreme forms, medium and winter-leafed forms are also sometimes distinguished. In the northeastern part of the *Q. robur* range, the bud burst phenological varieties are practically absent, but varieties differed by the terms of autumn lead fall were clearly distinguished [159]. The difference between the two bud burst forms or varieties in growing degree units (at the base temperature of 5 °C) is more than 15 °C according to [160] and up to 35 °C according to [161]. The difference in the time of leafing onset between the early and late forms ranges from 2 to 4 weeks [162,163,164,165]. As shown by studies of the genetic determination of phenological forms in oak, these forms separated due to a mismatch in their flowering periods [155,166]. Thus, the gene flow between them is limited [163], and their genetic differences are confirmed by molecular studies [167,168].

According to the phenological structure of the population, most trees maintain the same tendency from year to year, i.e., they remain in the same phenological group (designated as “early”, “middle”, and “late”) or change it by one phenological level, which indicates a genetic influence on the manifestation of this trait. Since these populations are located in similar environmental conditions, the differences between them can be considered as a consequence of intraspecific variability of *Q. robur* and the genetic structure of the population [169]. The phenological forms within the species *Q. robur* differ in the timing of leafing. Significant differences in vegetation timing requirements were found between the three flowering phase phenoforms, early, intermediate, and late [170].

In the forests of Lekenik and Otok in Croatia, local environmental factors influenced the timing and duration of *Q. robur* phenophases, contributing to variability in its morphological and physiological traits [171]. Variations in the start and end of flowering phases were influenced by both genetic makeup and environmental effects. Specifically, the onset of leaf phenophase development in *Q. robur* showed a notable difference of 42 days between early and late leaf-developing phenotypes, although no such differences were observed in reproductive phenophases. The timing of early and late leaf flushing in *Q. robur* was primarily determined by genetic factors, with environmental factors playing a lesser role.

Studies of oak phenoforms under similar soil and moisture conditions revealed no consistent association between leafing time and factors such as tree age, size, or stand position, suggesting that phenological variation may be genetically driven [172]. Comparative research on the long-term dynamics of growth and leaf phenology, xylogenesis in the two phenological forms, and their responses to meteorological factors show an absence of uniform climatic response. Instead, individual patterns in leaf and xylem development point to substantial genetic variability [173,174,175,176,177].

Significant differences in leaf phenology and xylogenesis timing were found among individual trees in *Q. robur* [174]. The smallest differences in wood formation between trees were observed at the beginning of the vegetation period, when the first earlywood vessels were detected (9 days). High seasonal variability in the number of cambial cell layers was observed. Differences in the timing of cambial activity cessation and xylogenesis amounted up to a month. Oak tree ring width and the size and number of early wood vessels may not be sensitive indicators of early spring temperature and spring defoliation; the lack of a relationship between leaf phenophases and xylogenesis, as well as phenological variability, may be the reasons for the lack of a clear climate model observed in [174].

The phenological forms are particularly noteworthy due to their differing responses to growth conditions, extreme climatic factors, and pest outbreaks. Most researchers observe that late-forming trees have a shorter growing season, are not affected by spring frosts, grow faster, produce higher-quality wood, and are more resistant to oak forest pests, including powdery mildew (*Erysiphe alphitoides*), *phytophthora*, lace bug (*Corythucha arcuata*), and various species of gall wasps (genus *Cynips*) [153,156,166,176].

The crown height of the early form is 15–20% higher than that of the late one [178,179]. In late-forming stands, there are usually more trees with valuable economic traits [179], the so-called “plus trees” [180,181]. Although young trees of the early form grow faster [177,179], the growth energy of the late phenological form increases with age [156,182].

Local growth conditions affect the growth rate of both phenological forms [162,181], but air temperatures have the most significant impact on leafing [160,183]. In addition to growth energy and leafing timing, morphological differences are also observed: the late form typically has straight, fully woody trunks and high, raised crowns, while the early form has wide, spreading crowns and curved trunks [166,184]. In humid soils with frost risks, the late form has an advantage over the early form. In drier conditions, the early form is less affected by spring frosts and outperforms the late form in growth rate. It is believed to better utilize favorable spring moisture conditions but is more susceptible to defoliation from late frosts and insects. Its earlier start to the growing season allows it to begin growth before summer droughts set in [164,173,185]. In contrast, the late form avoids the spring defoliation risk from frost and insects, with its growth more concentrated in the summer due to a delayed start of the growing season, making it more vulnerable to summer droughts. As a result, it tends to occur in more humid soils [176,185,186]. Understanding the biological and ecological traits of these phenological forms is crucial from a silvicultural standpoint, as selecting the wrong oak phenological form for specific moisture conditions can lead to forest management failures and oak stand decline [153,185]. Individual variability in the onset of individual phenophases or their duration influence the degree of plant resistance to herbivores and pathogens. *Q. robur* with an earlier onset of leaf fall are more susceptible to defoliators, since there is synchronization between the phenophase and the defoliator development cycle [164,187,188].

Early and late phenological forms of *Q. robur* were studied on healthy oak trees in permanent sample plots in wet, dry, and very dry oak stands grown on different soil types in Shipov Forest, Voronezh Region, Russia using dendroclimatic analysis [189]. The late phenological-form trees showed higher radial growth rates on wet sites than the early phenological forms, which had an annual radial growth that was less stable and more variable compared with the late phenological forms. For both phenoforms, the key factors influencing radial growth are composite indicators that reflect the balance between temperature and moisture, such as Selyaninov’s hydrothermal coefficient and Lang’s rain factor. Generally, the periods of minimal radial growth occurred simultaneously on both dry and wet sites, while maximum growth periods were linked to years with high water availability [189].

In conclusion, studies of *Q. robur* phenology reveal significant intraspecific variation in traits like leafing, flowering, and vegetative timing, which are influenced by genetic and environmental factors. Early and late phenological forms of oak differ in growth patterns, wood quality, and resistance to pests and climatic stress, with genetic determination playing a key role in phenophase timing and adaptation. These differences are vital for forest management, as selecting appropriate phenological forms can optimize resilience and growth under specific site conditions. Oak populations exhibit polycyclism, interannual variations in acorn production, and unique adaptations to local environmental factors. Late phenological forms are more resistant to frost and pests, while early forms take advantage of favorable spring conditions but are more prone to late frosts and defoliators. These insights inform conservation and breeding strategies, ensuring sustainable forest management in diverse climatic contexts.

## 10. Phylogenetics and Phylogeography of *Q. robur*

Based on 9549 conserved orthologous sequences a simplified phylogenic tree places oaks in the group of Fabids (Eurosids I) between peach and poplar genomes (Figure 3 in [140]. *Quercus* is a species-rich genus with a mostly Northern Hemisphere distribution of around 435 species divided into two subgenera (*Quercus* or “white oaks” and *Cerris* or “red or black oaks”) and eight sections: five in subgenus *Quercus–Quercus* (white oaks in North America and Eurasia), *Mesobalanus* (Central and Eastern European), *Ponticae* (mainly in Western Asian), *Protobalanus* (golden oaks in the Southwestern United States and Northern Mexico), *Virentes* (live oaks common in the Southeastern U.S.), and three in subgenus *Cerris*–*Cerris* (Turkey oaks mostly in Eurasia), *Ilex* (Holly oaks in the Mediterranean), *Lobatae* (red oaks in primarily distributed in North America) [190,191,192]. It was shown based on genetic data that oaks have tighter macrosynteny with and are more closely related to chestnuts than to beeches [193].

Genetic data on 260 oak species have shown for the first time how these species have varied across the continents of the Northern Hemisphere and that their rates of diversification have increased in response to migration to new habitats [192]. The oak gene phylogenies are highly reticulate and reflect histories of introgression, lineage sorting, and divergence [192]. Fossil data and restriction-site associated DNA sequencing (RAD-seq) for 632 individuals representing nearly 250 oak species were used in [192] to infer a time-calibrated phylogeny of the world’s oaks and to investigate global patterns of oak diversity and test the hypothesis that there are regions of the oak genome that are informative about phylogeny. Oak lineages have diversified across different geographic regions, followed by ecological divergence within those regions, in both the Americas and Eurasia. Approximately 60% of oak diversity can be traced to four clades that underwent increases in net diversification, likely driven by climatic changes or ecological opportunities. While the phylogeny is strongly supported, it contrasts with significant genomic heterogeneity in phylogenetic signals and introgression. Oaks are phylogenomic mosaics, and their diversity may actually be influenced by the gene flow that shapes the oak genome. Oaks of Eurasia point to the important role of ecological and morphological convergence among unrelated oaks. The phylogeny of the Eurasian white oaks represented by 23 of the estimated 25 white oaks in the Roburoid clade of section *Quercus*, including *Q. robur*, has been addressed in detail with the strongest sampling of white oak species up to date. They concluded a northern temperate clade. The increase in diversification rate in the Roburoids with divergence within clades and geographic regions is in agreement with their ecological diversity, ranging from lowland swamp to Mediterranean scrub, and from mesic lowland forests to subalpine timberline. The European Roburoids are not readily diagnosable morphologically, and the morphological and ecological convergence among clades has led to taxonomic confusion. This study demonstrates that across the genus, ecological diversification within clades has shaped diversification and that there is not a single genome part that can discriminate all the oak species [192]. A dated phylogeny of oaks shows high diversification rates since the Pliocene in sections *Quercus* and *Lobatae* [192].

The geographic distribution throughout Europe of each of 32 chloroplast DNA variants belonging to eight white oak species sampled from 2613 populations including pedunculated oak is presented in [87]. These distributions, together with the available palynological information, inferred colonization routes out of the glacial period refugia. In western Europe, movements out of the Iberian and Italian Peninsulas are particularly evident. Separate refugia were also found in the Eastern Balkans, while the western part of the peninsula showed similarities with Italy. These movements, which likely involved the exchange of haplotypes between refugia during the current interglacial period and possibly during earlier glacial cycles, suggest that phylogenetically divergent haplotypes often followed similar colonization routes, somewhat limiting the phylogeographic structure. Geographic disjunctions in the current distribution of haplotypes are also apparent, likely caused by rapid climatic shifts at the end of the glacial period (notably the Younger Dryas cold phase), which led to range contractions following an earlier warm period when oak first expanded from its primary refugia. This cold phase was followed by another expansion period at the start of the Holocene, which in some cases involved “secondary” refugia. These short climate oscillations likely caused a partial reshuffling of haplotype distribution. The data also suggest early associations between haplotypes and oak species, although extensive introgression among species has largely obscured this pattern. This implies that colonization routes may have initially been shaped by the ecological traits of the species hosting each chloroplast variant. For instance, it suggests that two oak species in the north of the Iberian Peninsula (*Q. petraea* and *Q. pubescens*) are recent post-glacial immigrants. When considered together, the conclusions drawn from molecular data and fossil pollen data regarding the location of glacial refugia and colonization routes appear to be largely compatible and complementary [86].

Oaks have a remarkable evolutionary history [194]. Genomic data on early and recent oak evolution reveal evolutionary mechanisms that allowed oaks to colonize the Northern Hemisphere [195]. It suggested that Roburoid oaks arose as a result of migration of the oaks in the North American section *Alba* into Eurasia and introgressive hybridization with European species of the section *Pontica*.

Geographic structure of chloroplast DNA variation studied in 42 populations of *Q. robur* in the European part of Russia, Belarus, Poland, Ukraine, the Urals, and the Caucasus demonstrates sharp differences between western and eastern populations [81]. The authors concluded that this outcome is due to postglacial colonization by both species from distinct, long-isolated refugia, some of which were located in the eastern part of the studied areas. In the Urals, the differentiation of oak between the southern regions (Ural River and Belaya River basins) and the more northern areas (Ufa River basin and the Middle Urals) can likely be attributed to different historical colonization patterns of these regions by oak [81].

Genetic differentiation was studied in 41 populations (755 trees) of *Q. robur* and six populations (109 trees) of *Q. petraea* in Northeastern Europe, the Balkans, Crimea, and the Caucasus using 14 nuclear microsatellite loci (nSSR) and compared with the previously obtained chloroplast DNA data [103]. A low level of nuclear DNA introgression of *Q. petraea* in *Q. robur* was revealed. No significant influence of *Q. petraea* on the geographic structure of *Q. robur* diversity was found. High differentiation in nuclear markers between populations of *Q. robur* from different geographical regions and within the Caucasus was obtained. The largest difference was observed between Caucasian populations and the populations of Northeastern Europe. In the northern part of Eastern Europe, a weak trend from east to west was observed based on nuclear markers, in contrast to the chloroplast DNA data, which revealed a sharp distinction between the populations in the eastern Russian Plain and Urals and those in the western regions due to the absence of shared haplotypes. Populations in the Southeastern Balkans differed from those in northeastern Europe, and the ABC estimate of divergence time (910 generations) significantly exceeded the age of the Last Glacial Maximum, ruling out the involvement of the Southeastern Balkans in the recolonization of Eastern Europe after the last glaciation. In the Caucasus, the structure of nuclear DNA generally aligned with the phylogeography of chloroplast DNA and morphological data. There was a clear differentiation between Western and Eastern Caucasian populations based on nuclear markers. A mix of the Balkan and West Caucasian clusters was identified, which, along with chloroplast DNA data, suggests their origin from multiple migration events. This group of populations contained only West Caucasian and Crimean endemic chloroplast haplotypes, without Balkan haplotypes, indicating gene introgression from the Balkan cluster into the Crimean and West Caucasian populations via pollen flow, without seed transfer [103].

Summarizing, oak species, comprising around 435 globally distributed species, exhibit significant genetic and evolutionary diversity. Phylogenetic studies reveal that oaks share closer relationships with chestnuts than beeches and that diversification was driven by ecological adaptations to new habitats. This diversification is evident in the distinct clades and ecological niches occupied by oaks in Eurasia and the Americas. Postglacial colonization patterns, informed by chloroplast DNA and fossil pollen data, highlight distinct refugia and migration routes shaped by climatic shifts, such as the Younger Dryas. Genetic analyses also show extensive introgression among oak species, resulting in complex phylogenomic mosaics. In Europe, geographic differentiation in oak populations, particularly between western and eastern regions, reflects historical migrations and ecological conditions. Studies indicate that hybridization and genetic flow, influenced by both pollen and seed dispersal, have played a significant role in shaping oak diversity and distribution.

## 11. Response of *Q. robur* to Environmental Factors in Dendroecological and Dendrochronological Studies

Response and adaptation to environmental factors, identification and cataloguing of genes of adaptive significance remains an important step in understanding the evolutionary responses of *Q. robur* to selection pressures. The key factors implicated in oak decline and effective management of declining oaks include drought, pests and pathogens, but extreme frost, waterlogging, soil properties, land management, nitrogen pollution, heavy metal pollution, genetic predisposition, and mycorrhizal changes could also be involved in decline [196]. The amount that each main stressor contributes towards these declines and the “tipping points” are discussed below and are likely to differ between decline events, sites, and even individual trees.

### 11.1. Drought

In an experiment with scions grafted and vegetatively propagated from 40 pedunculate oaks older than 80 years from natural stands in the Republic of Croatia moderate drought stress significantly reduced net photosynthetic rate, stem diameter increment and height growth increment, but acorn mass was not affected [197]. Comparison of drought and nitrogen deficiency factors showed that moderate drought is a more dominant stress factor for photosynthesis and growth of *Q. robur*, but not for acorn development. Acorn mass decreased in both wet and dry years under suboptimal nitrogen nutrition [197].

It was demonstrated in another experiment that droughts increased the accumulation of osmolytes, such as proline and glycine betaine, as well as higher polyamines (spermidine and spermine) levels and decreased putrescine levels in *Q. robur* seedlings [198]. This study demonstrated also beneficial function of the ectomycorrhizal fungi, in particular *Scleroderma citrinum*, in reducing the effects of drought stress in pedunculate oak.

The response of oak trees to drought depends on its physiological status and phenological form. The most significant differences in the relative expression levels of candidate genes associated with drought tolerance were observed during the flowering period between phenoforms and between senescent and vital trees [199]. Three genes *wrky53*, *rd22*, and *sag21* showed upregulated expression pattern in senescent physiological groups, indicating their possible role in the coping mechanisms of oak in stressed environment.

Overall, *Q. robur* is considered a very drought-tolerant species and a relatively major beneficiary of climate change among broadleaf species [200].

### 11.2. Waterlogging

The radial growth of *Q. robur* depends on precipitation at the beginning of the growing season, and from late spring until the end of the growing season it increasingly depends on the groundwater level [201]. These authors also revealed that radial growth during the *Q. robur* vegetation period depends on various water sources: in spring, growth is mainly driven by precipitation, while in summer and autumn, groundwater levels play a more critical role [202]. Overall, this study highlights the considerable threat that both groundwater changes and the increasing frequency of drought events pose to floodplain forests. The oak’s dependence on water bodies and the proximity of groundwater increases their photosynthetic activity, with the lowest photosynthesis values being determined in the area furthest from the water source [203].

The *Q. robur* adapts to waterlogged soil by changing the anatomical features of the wood—a general increase in the number of vessels and the width of the annual ring [204]. The *Q. robur* has the ability to quickly regenerate the root system after waterlogging (or flooding), which allows it to survive in hydromorphic soils. It is assumed that the *Q. robur* is able to preserve its carbohydrate reserves until the growing conditions become more favorable [205].

Resistance to waterlogging in *Q. robur* is determined by molecular mechanisms of formation of a radial barrier against oxygen loss in the roots, similar to species resistant to waterlogging due to increased biosynthesis of suberin [206].

Adequate water availability during the spring months promotes the development of early wood, including the differentiation of spring vessels, as confirmed by the study of a floodplain *Q. robur* population in Czech Republic [207]. The overall wood formation depends not only on the specific conditions of the growing season but also significantly on the tree’s local position within the stand.

The radial growth of *Q. robur* (pedunculate oak) relies on precipitation early in the growing season and increasingly on groundwater levels from late spring onward. Its growth during the vegetation period is driven by various water sources: precipitation in spring and groundwater in summer and autumn, making it highly vulnerable to changes in groundwater availability and drought frequency. Adaptations to waterlogged soils include anatomical changes like increased vessel numbers and wider annual rings, rapid root regeneration, and molecular mechanisms that enhance resistance, such as forming oxygen barriers in roots. Local stand conditions also influence overall wood formation. These traits highlight the oak’s ecological resilience but underline its sensitivity to hydrological changes.

### 11.3. Geographic Origin and Cultures, and Provenance Tests

The large area of natural growth of *Q. robur* indicates its wide ecological amplitude. Its populations adapt to a variety of different microhabitats, promoting inter- and intra-population variability. A study of biomass accumulation depending on environmental conditions and origin showed a greater influence of genetic characteristics of populations, although leaf parameters are more dependent on micro-environments of growth [208].

The mother trees origin affects the morphological characteristics of *Q. robur* seedlings [209]. A high degree of variation has been observed for root collar diameter, height, weight of above-ground part, root weight, Roller’s sturdiness coefficient, and seedlings quality index. The high degree of correlation was observed between root weight and above-ground part weight. Provenance trial and common garden experiments in Belgium and Denmark demonstrated that the environment of origin influenced the bud phenology of seedlings, and this provenance effect was dependent on the seedlings’ growing environment. Furthermore, the results suggested that the impact of warming may vary between provenances, and that the environmental history of previous generations is likely to affect the response of tree seedlings [210].

Variability of oak is often associated with the frequency of interspecific hybridization, which occurs as a result of the absence of a phenological barrier during the flowering period between *Q. robur* and *Q. petraea*. Locality and climatic conditions have a dominant effect on plant growth and development, i.e., on nutrition and absorption of nutrients. The observed variability in plant adaptability is manifested in changes in the size and weight of leaves, the number of stomata, and may be a consequence of species adaptation [155].

There is significant variation in the physiological response of *Q. robur* trees of different origins to heat stress, and selection of offspring from origins with a more pronounced adaptive response is possible [211]. Populations of *Q. robur* with similar morphological and physiological characteristics could be combined into groups in accordance with the ecological zoning of vegetation in Bosnia and Herzegovina; it was suggested that it is possible to use reproductive material within these groups of regions for reforestation and, with additional study, an exchange between certain regions is also possible [212]. Genetic analysis is currently used to select the origins with the best production potential; by selecting the best origins of *Q. robur* and propagating them, it is possible to achieve a significant increase in the height and thickness of the plants, i.e., the yield of wood pulp [213].

Summarizing, *Q. robur* exhibits a wide ecological amplitude, adapting to diverse microhabitats and demonstrating high inter- and intra-population variability. Genetic characteristics primarily influence biomass accumulation, while leaf traits are more affected by local microenvironments. Seedling traits, including height, root weight, and sturdiness, vary significantly depending on the mother tree’s origin. Provenance and environmental history also affect seedling bud phenology and responses to warming. Hybridization with *Q. petraea* and environmental conditions significantly shape variability in growth and adaptability, influencing traits like leaf size and stomatal number. Genetic selection of favorable origins enhances growth and productivity, offering potential for improved reforestation efforts and wood yield.

### 11.4. Climate Change, Temperature, and Precipitation

Predictive models show that climate change will negatively affect the ecological niche of *Q. robur* in the future. For example, in Croatia, the model predicts an increase in the minimum temperature in the coldest month and the maximum temperature in the warmest month, as well as a decrease in precipitation in both the driest and wettest months [214]. Climate change is seen as a leading factor in oak decline in Southern Sweden according to a simple model linking climatic variation, site-level condition, and the risk or proportion of trees affected by the decline [215].

Poor winter hardiness is thought to limit oak cultivation in northern latitudes and set a limit to the northward expansion of *Q. robur*, but high intra-population variability offers opportunities for the development of more frost-resistant genotypes [216]. It is interesting that the results of a study of oak regeneration in Sweden showed a lack of latitudinal association with oak regeneration, in contrast to the assumption of decline towards the range margin [217]. Contrary to the hypothesis that oak regeneration would decrease at the range margin, these results did not support this notion. This suggests the possibility of a future northward expansion of oak populations. The study also found a positive correlation between oak regeneration and stand age, while higher nitrogen and ground moisture levels were inversely related to regeneration. The age-dependent positive effect on recruitment implies that species recruitment dynamics within forests may be influenced by age-related factors in the tree community, with important implications for forestry and conservation management. Notably, the successful natural regeneration of the introduced *Q. rubra* highlights its adaptation to the Swedish climate and forests. This study represents the first large-scale analysis of oak regeneration across multiple oak species in Sweden [217].

The spring bud break phase of *Q. robur* depends on both genetic characteristics (phenoform) and climatic factors. It was demonstrated that for all three studied flushing groups (early, intermediate, and late flushing groups, respectively) insolation, and the day of year were statistically significant predictors for growing degree days (GDD) [170]. Insolation was demonstrated to be the primary factor with the largest influence on the GDD values. GDD model was univariate for the early (GDD_early_ = −27.651 + 0.539 × insolation) and intermediate (GDD_inter_ = −48.084 + 0.690 × insolation) flushing groups and the multivariate model (GDD_late_ = −237.839 + 0.559 × insolation + 2.479 × day of year) for the late flushing group. To break dormancy, chilling needs to fall within the range of ± 40 chilling units. If the values are outside this range, the trees will require significantly higher forcing temperatures [170].

The study by Firmat et al. [218] showed that leaf phenology traits depend primarily on the plastic variability exhibited by a tree during its life. Both spring and autumn (bud break and senescence, respectively) phenological periods were involved in local adaptation, but bud break was likely the trait most susceptible to climate change-induced selection.

The increase in spring temperatures observed over the last decade significantly increases the reproductive capacity (formation of acorns) of temperate oaks. With warming, not only the number but also the mass of acorns increases [219,220], while the amount of precipitation does not significantly affect the formation of acorns [219].

In general, there was an increase in *Q*. *petraea* and *Q*. *robur* vegetative and reproductive growth in recent decades in central Europe. Along with rising temperatures, higher carbon dioxide levels in the atmosphere may also enhance tree growth in certain species, while increased nitrogen (N) deposition has been shown to boost forest growth and carbon sequestration in Europe. Thus, global warming may promote the proliferation of oak in temperate ecosystems [219].

In conclusion, *Q. robur* is increasingly impacted by climate change, with models predicting reduced suitability in its ecological niche due to rising temperatures and altered precipitation patterns. While poor winter hardiness limits its northern expansion, high genetic variability offers potential for developing frost-resistant genotypes. Contrary to expectations, oak regeneration in Sweden showed no decline at its range margin and may even indicate potential for northward expansion, particularly in older stands with less nitrogen and ground moisture. Phenological traits, like bud break and senescence, are influenced by genetic and climatic factors, with spring bud break strongly linked to insolation. Warmer spring temperatures over the last decade have enhanced acorn production, both in number and mass. However, premature spring bud break and delayed fall dormancy make oaks vulnerable to the late and early frost episodes that have become more frequent in the past years due to the irregular climate. While precipitation has minimal impact on acorn formation, global warming, increased atmospheric CO_2_, and nitrogen deposition may drive growth and reproductive success, potentially boosting oak proliferation in temperate regions.

### 11.5. Climate Response in Archaeological, Dendrochronological, and Genetic Studies

Signature years of oak growth across northern Europe show consistent patterns tied to climatic anomalies [221]. Growth variations correspond to pan-European differences in soil moisture, rainfall, and temperature, with enhanced growth linked to extended soil moisture availability and reduced growth tied to lower temperatures and dry conditions. These anomalies are connected to large-scale atmospheric circulation perturbations, particularly involving the circumpolar vortex and the Arctic Oscillation. A 2000-year record indicates decadal and centennial variations in the frequency of these signature years, providing a potential proxy for historical climate data [221]. Despite uncertainties about the provenance of timber records, these findings support the idea that regional archaeological chronologies contain valuable climatic information, useful for reconstructing past climate patterns.

Using 953 oak tree-ring samples from Central Germany (spanning AD 996–2005), Büntgen et al. [222] reconstructed drought variability and identified that tree rings explain 18–70% of inter-annual to decadal summer drought variance. Common extreme drought and wet years include 1934, 1959, 1996, and 1958, 1966, 1967, respectively. Positive correlations were found between regional drought indices and European hydroclimate patterns, with increased mid-tropospheric geopotential height anomalies over the British Isles linked to drought. Conversely, negative anomalies over Western Europe facilitated wet summers through moist air advection [222]. The authors concluded that while these findings illuminate synoptic-scale drought dynamics, long-term trends remain uncertain.

A study of the intra-annual growth patterns of beech (*Fagus sylvatica*) and oak (*Q. robur*) trees on a sandy site in the Netherlands during 2003 and 2004 found that wood formation in both species halted during the summer drought of 2003, but beech demonstrated a capacity to recover afterward, unlike oak [223]. These findings highlight species-specific differences in drought resilience, which are crucial for assessing tree susceptibility to climate change and understanding growth responses at fine temporal scales.

Sohar et al. [224] examined how climate influences the radial growth of *Q. robur* at its northern distribution limit in Estonia, using tree-ring data from 162 oaks across three regions: Western, Northeastern, and Southeastern Estonia. The growth responses varied by region due to local soil and climate conditions. Oaks in Western Estonia, growing on shallow soils, were positively influenced by summer precipitation, while northeastern oaks, on deeper soils, were favored by June temperatures. Southeastern oaks depended on both July precipitation and temperature. Although the regional growth patterns aligned broadly with adjacent areas, local variations were significant, particularly in extreme climate years (pointer years).

To adapt future management practices for *Q. robur* and *Q. petraea* in Southern England and Northern France under environmental change, dendrochronological analysis of their growth patterns from 1900 to 2010 was conducted [225]. The study found that oak growth rates have significantly increased over the 20th century, demonstrating resilience to climatic and environmental challenges. Oaks in monoculture stands generally outperformed those in mixed stands in Southern England’s New Forest, but no such difference was observed in Thetford Forest. Pointer years of extreme growth were often region-specific, indicating that local factors, rather than broad climatic trends, primarily influence growth patterns.

Perkins et al. [226] examined the growth responses of *Q. petraea* and *Q. robur* under varying climatic conditions in their natural ranges in Southern Germany and Northeast Italy and beyond their natural range in South Africa. Tree-ring analyses revealed that while long-term growth trends were consistent across sites, growth levels were higher in warmer and drier climates than in temperate zones. Oaks displayed greater-than-expected growth in recent decades, though a slight decline has been observed recently. Short-term responses to drought events showed reduced growth 2 to 3 years after drought, with oaks in South Africa already experiencing climate conditions predicted for Europe’s future. The study highlights the implications of these growth behaviors for tree species selection in the context of climate change.

The tree-ring chronology obtained for 20 trees of *Q. robur* growing in Donetsk Region (the steppe zone in Eastern Europe) is strongly correlated with local spring and summer precipitation, and local temperatures in April, June, and July. Dynamic correlation analysis indicates that the relationships between oak growth and late winter and early spring temperatures, and between oak growth and February and August precipitation, have changed over the past 80 years. These data suggest that warming has led to both advances in oak phenology and changes in early spring climatic conditions [227].

Matveev et al. [228] analyzed 30 years of climate dynamics in the Voronezh region compared to historical norms, focusing on their effects on the radial growth of Scots pine (*Pinus sylvestris*) and *Q. robur*) in the Voronezh region (Southern Russia). They found the strong influence of April and May precipitation on tree growth. Using mathematical models, the study forecasts potential changes in growth and productivity for these species in response to ongoing climate variability.

The greatest influence on the radial growth of *Q. robur* in western and northeastern Poland was exerted by spring precipitation in the year preceding the formation of the annual ring, and the temperatures of the previous and current springs, when the trees formed the growth [229]. The response of forest stands to climatic factors depends on their age. In young forest stands, a contradictory response was observed, in contrast to older ones.

A study of the climatic response in *Q. robur* populations in the Southern Urals showed that the response to monthly temperatures in radial increment was more pronounced in the mountain stands, while it was absent in the Bashkir Cis–Urals [230]. On the contrary, in the latter part of the region, trees were more sensitive to precipitation. In some cases, the considered climatic factors had opposite effects on different stands. Precipitation in winter (December and January), May, and summer (June and July) were especially important for the radial increment of the species. In the mountain stands, significant negative correlations were observed between this parameter and the air temperature of October of the previous year, March, April, May, and July of the current year. Thus, the climatic response of English oak has regional features and depends on the biological characteristics of the analyzed individuals/populations (age, origin).

Rellstab et al. [231] used a pooled amplicon sequencing of 94 genes in 71 populations of *Q. petraea*, *Q. pubescens*, and *Q. robur* in Switzerland to genotype ~3500 SNPs and associate their variation with abiotic factors related to local topography, historical climate, and soil characteristics. They also tried to predict the fate of these species under the scenario of climate change. They identified several SNPs and genes likely involved in local adaptation and pinpointed the environmental factors that may be driving this process. They then integrated the allele frequency data of these candidate SNPs with current and future climate data to assess the potential adaptability of the oak populations and species under projected climate change. In total, they found 545 significant abiotic factors-SNP associations for 181 SNPs in 68 genes in *Q. robur*. In *Q. robur*, in this order, humidity, number of precipitation days in summer, annual precipitation, continentality, mean annual temperature, index for average summer drought, and clay content were the factors most often associated with SNPs. In average, populations of *Q. robur* were modelled to experience the smallest increase in mean annual temperature. Risk of nonadaptedness (RONA) to future mean annual temperature and site water balance (precipitation minus evapotranspiration) was modelled, and for both environmental factors, the general linear mixed model revealed that RONA to the projected future climate varied significantly among species and populations within species. While *Q. robur* populations were predicted to be the most adaptable to changes in mean annual temperature, they were the least well-adapted to future changes in site water balance. As a result, *Q. robur*, typically found in warm, moist habitats, exhibited the lowest RONA to increased temperature and the highest RONA to reduced water availability in the future [231].

Notably, the plants’ response to global change can be rapid, even in long-lived species, like *Q. petraea*, which is a very close relative to *Q. robur* and displayed a genome-wide response to the Little Ice Age (~1650 AD) despite its ~50-year generation time [232]. Although *Q. petraea* is predicted to maintain its climatic niche at the northeastern cooler boundary, it is expected to produce wood at a slower rate due to the anticipated warmer climate in central Europe, with more frequent heatwaves and summer droughts [233].

Meanwhile, it seems that *Q. robur* has a significant adaptive potential to withstand the climatic stresses and adapt to climate change, although, perhaps, with some help via assisted migration [42,234,235]. For instance, the genetic diversity of 720 candidate genes associated with bud burst in *Q. robur* individuals sampled from six provenances in the provenance/family common garden trial of *Q. robur* established in Forest District Mogilica in Northwestern Poland and representing eight forest seed stands in Poland was explored using the sequence capture technique [236]. In total, 18,799 SNPs were genotyped in 720 candidate genes in 87 trees of *Q. robur* originated from six provenances. Using landscape genomic approaches, eight *F_ST_* outliers were identified and 781 unique SNPs in 389 genes associated and correlated with geography, climate, and phenotypic variables (individual/family spring and autumn phenology, family diameter at breast height (DBH), height, and survival) that are potentially involved in local adaptation. Then, vulnerable areas of the *Q. robur* distribution in Poland that are at risk from climate change were identified using a nonlinear multivariate model, Gradient Forests. The *Q. robur* populations in the eastern part of the analyzed geographical region are the most sensitive to climate change. Use of genomic resources and approaches to assess adaptive potential, divergence and introgression in oaks is nicely reviewed in [89].

The studies reviewed in this chapter highlights various climate and growth responses of oak species (mainly *Quercus robur* and *Quercus petraea*) across Europe and beyond. Key findings include the identification of specific climatic drivers, such as temperature, precipitation, and drought, influencing oak growth in different regions. Tree-ring data reveal that oak growth is sensitive to local climate variations, with long-term trends of resilience, though some regions show significant differences in how oaks respond to extreme weather events like droughts. Studies also suggest that oak populations have some adaptive potential to future climate changes, though species like *Q. robur* may face challenges due to reduced water availability. Furthermore, genetic studies indicate that oaks exhibit local adaptation to their environment, with varying degrees of risk under future climate scenarios. Climate models predict that *Q. robur* may struggle with water availability, while *Q. petraea* may cope better with warmer temperatures but at slower growth rates. The genetic diversity of oak populations could play a crucial role in their ability to adapt to these changes and should be maintained to guarantee oak species sustainability.

## 12. Factors of Resilience to Environmental Factors in Oaks and Their Longevity

An important feature of *Quercus* species that contributes to their stability and distribution is the presence of climatypes. Using North American oaks as an example, it has been shown that species are differentiated within lines by sets of functional traits that show correlated evolution and adaptation to contrasting habitats [237]. Climatypes have multidirectional indicators associated with resistance to various factors. For example, areas with frequent and severe fires are characterized by oaks with a high ability to grow shoots from rhizomes and increased bark thickness [238]. The altitudinal gradient also affects physiological and morphological features [239,240]. Thus, depending on the conditions of the territory, it is necessary to select the most suitable climate types. Recent studies on the drought resistance of *Quercus* species, including *Q. robur*, have shown that their resilience to drought stress involves a balance between mechanisms to prevent water deficiency and the ability to quickly restore water levels after stress. For example, research highlights that during drought, oaks exhibit reduced stomatal conductance and a decrease in water potential, which limits water loss from leaves and maintains internal hydration. Additionally, upon rehydration, physiological processes such as photosynthesis and enzyme activity rapidly return to normal, helping the plant recover efficiently. This combination of minimizing water loss and promoting rapid water uptake upon rehydration is essential for oaks to survive in fluctuating moisture environments [241,242,243]. Oak species respond to drought through increased antioxidant enzyme activity and osmotic solute accumulation, which help mitigate oxidative stress and maintain cell turgor under low water conditions [244,245]. These adaptations underscore how *Quercus* species balance both moisture retention in leaves and recovery to maintain growth and survival in dry conditions.

Lifespan probably correlates with resistance to stress and adverse environmental factors. Thus, forest communities whose framework consists of long-lived trees are apparently more resistant and adaptive to adverse external factors. Research in various fields of forest science reveals features that may be associated with a long lifespan of trees. It was theorized that the “chemostat effect” model observed in bacterial colonies that persist for several generations and accumulate neutral mutations is applicable to long-lived woody plants [246]. The extent of mutational load in populations of long-lived plants depends on the origin and accumulation of mutations. If mutation rate is measured as mutations per cell division, per growing season, or another similar biological time unit, the overall frequency of mutant cells will rise as the plant ages (the aforementioned “chemostat effect”). Thus a 20-year-old tree should have a lower mutation frequency in its sex cells than a 200-year-old tree of the same species. Based on the fact that apical meristems undergo about five divisions of initials during the growing season, Klekowski suggested that approximately 5% of the initials would be mutant in 100-year-old trees, 10%—in 200-year-old trees and so on; tree branches are considered as stem cell lines [246]. During iterative branching, the number of branches grows exponentially, while the number of cell divisions rises linearly [247]. Additionally, computational modeling demonstrates that the arrangement of stem cells and the positioning of axillary meristems help distribute somatic mutations across the main shoot, preventing their fixation and promoting genetic diversity. These features slow down “Muller’s ratchet” (accumulate deleterious mutations in an irreversible manner) and thereby extend lifespan.

This theory of accumulation of somatic mutations was confirmed for the genus *Quercus* by Plomion et al. [78]. The emergence of new gene variations is associated with meiotic mutations that occur during sexual reproduction. Somatic mutations that arise in apical meristems and can be transmitted to generative tissues and offspring were also found in oak. Therefore, somatic mutations along with meiosis can increase the genetic diversity of long-lived trees [78], which may also indicate the contribution of mutational load to adaptation, in particular with regard to protection against new pests and pathogens. The accumulation of somatic mutations in resistance genes was shown also for another long-lived tree—*Eucalyptus* [248].

Moreover, increased lifespan largely depends on the immunity of trees to infectious organisms. Based on the analysis of the *O. robur* genome, Plomion et al. [78] showed that disease-resistance genes have multiple tandem duplicates, thus increasing the number of copies of an important gene aimed at preventing damage caused by pathogens and therefore the resistance of the organism as a whole. It is assumed that the immune system makes a significant contribution to the survival of long-lived plants for several centuries and leads to adaptive plasticity [78,249]. According to [249], tree defense and longevity are based on three genomic approaches: (1) gene numbers, (2) genomic architecture, and (3) mutation loads accumulated over long life spans. The genomic architecture is expressed in the clustering of genes and their repetition, a high number of transposable elements. Mainly, the genes of the metabolic pathway and R-resistance genes are clustered.

Such long-lived sessile organisms as trees must persist in the face of a wide range of abiotic and biotic threats throughout their life span. A consequence of the long life span of trees is the accumulation of somatic mutations during mitotic divisions of stem cells present in shoot apical meristems. Empirical approaches and modeling have shown that intra-organism genetic heterogeneity provides advantages in the fight against short-lived pests and pathogens due to the combination of intra-organism phenotypes. In the oak example, we see the accumulation and transmission of somatic mutations and the expansion of tree disease-resistance gene families [78]. However, for old-growth trees, low levels of mutations in meristems were shown. Schmid-Siegert et al. [250] sequenced the genome of two terminal branches from a 234-year-old *Q. robur* tree and identified several fixed somatic single-nucleotide variants. These variants could be traced through nested sectors of younger branches, revealing a sequential pattern. Their findings suggest that the stem cells of shoot meristems in trees are effectively shielded from the accumulation of mutations. They found that oaks protect their bud meristems from ultraviolet radiation with multilayered leaf-like structures, potentially reducing the likelihood of ultraviolet mutagenesis [250]. This can contribute into maintenance of tree health and explain the life span that can reach several hundred years, up to almost half a century in *Q. robur* [251]. Thus, long-lived trees, such as *Q. robur*, have mechanisms for both protection against somatic mutations and fixing the emerging “useful” mutations to combat pathogens. Different genetic mechanisms of aging in trees are reviewed also in [252,253,254,255,256].

Ianbaev et al. [257] suggest that maintaining a high level of genetic variability in *Q. robur* populations is also the basis for their resistance to environmental factors in the context of global climate change. The authors suggest that this may be due to the efficiency of genetically realized pollen flow, including between remote stands. Based on 412 SNPs, high intrapopulation genetic variability was shown in *Q. robur* populations in the Republic of Bashkortostan (Russia) growing in significantly different rainfall regimes and amounts, mainly by months of the growing season [257]. Thus, it is important to note the need to maintain high genetic diversity in *Q. robur* populations, which is necessary for adaptation to changing environmental conditions, through maintaining effective gene flow between remote stands, including through the creation of green belts and promoting long-distance gene exchange via assisted migration.

Oak species, such as *Q. robur*, exhibit “climatypes” that help them adapt to various environmental conditions. These climatypes, which include adaptive mechanisms for water retention, rapid recovery from drought stress, and such adaptive traits as increased resilience to oxidative damage, increased bark thickness, and rhizome sprouting in fire-prone areas, contribute to drought resistance through a balance of water retention and rapid recovery. Long-lived trees like oaks also accumulate useful somatic mutations over time, which may help them adapt to environmental stresses, including pests and pathogens. Their longevity is further supported by genetic mechanisms that protect stem cells from harmful mutations. Furthermore, oaks possess enhanced immunity through gene duplication and efficient genetic diversity maintenance, which supports their survival over centuries. Maintaining genetic diversity within oak populations is crucial for adaptation to climate change, as it enhances their resilience to fluctuating environmental conditions, highlighting the need to conserve genetic diversity in oak populations.

## 13. Plus Trees and Main Breeding Traits of *Q. robur*

Traditional breeding of *Q. robur* was carried out by selection of stands (origins, plus stands, etc.), individual selection (selection by progeny), and crossing or hybridization [258,259]. Breeding is carried out in two directions toward economically valuable traits and adaptation to changing climatic parameters. In order to obtain maximum productivity of valuable timber, selection markers are focused on morphological traits of the trunk, physical, and mechanical properties of wood. In addition, selection of origins based on the progeny with a properly formed habitus is possible, which is based on the identified correlation between root mass and above-ground part mass and the heritability of this relationship. This feature may facilitate the selection of suitable reproductive material sources for restoring *Q. robur* forests within a particular region of distribution [169]. For maintaining populations and preventing degradation of oak forests, selection is aimed at choosing maternal populations that show adaptive growth in changing conditions, and in which collection of standard seed material is possible. Forest tree breeding and silviculture of *Q. robur* need to take into account large genetic multiplicities, to manage *in situ* maintenance of numerous and sufficiently large, locally adapted stands [260].

In order to obtain maximum productivity of valuable timber, selection markers are focused on morphological features of the trunk, physical, and mechanical properties of the wood. In addition, selection of origins by progeny with a correctly formed habitus is possible, which is based on the identified correlation between the mass of the root and the mass of the above-ground part and the heritability of this relationship. This feature may facilitate the selection of suitable reproductive material sources for regenerating *Q. robur* forests within particular region of distribution [209].

Kostrikin et al. [261] developed criteria for selection of plus stands. For an ideal variety of forest tree species, including *Q. robur*, it is proposed to use the following main parameters: productivity, variability, genetic determination, wood characteristics. They suggest classifying stands as “plus stands” if their forest density is between 0.7 and 0.9, more than 35% of the trees have commercial-quality wood, and the plot contains at least one plus tree per two hectares. It is noted that under the externally outstanding phenotype there are hidden quality features of the different value, with their variation from the lowest (wood density) to the highest (sapwood width) level [262]. Thus, it is impossible to limit oneself exclusively to phenotypic parameters; it is necessary to supplement the characteristics of plus trees with features of macro- and microstructure, indicators of physical and mechanical properties. In Japan, England, Sweden, Bulgaria, and the USA, breeding is also based on wood density characteristics.

The success of selection activities depends on the comprehensiveness of the approach to selection and determination of the leading group of traits, as well as the assessment of heritability [258]. The most valuable for selection are plus trees. The following can act as “plus trees”:

- trees-phenomena;
- trees-veterans;
- record-breaking best in technical and economic terms and at the same time highly viable;
- fast-growing;
- straight-trunked;
- knot-free;
- possessing the best wood qualities;
- undamaged by rot, harmful insects, frost and other factors.

Plus trees represent a valuable state breeding resource for the improvement and reproduction of forests and their use in protective afforestation [263,264]. Using the example of the Krasnoye Forestry of the Vorontsovsky timber industry enterprise of the Voronezh Region in the Shipovaya oak grove (Russia), where the largest number of plus trees was found, criteria for the assessment and selection of plus oak stands have been developed for the purpose of harvesting selectively improved seeds [261].

Breeding and forestry programs are generally aimed at preserving oak stands. The current stage of forest management development should include measures both for selecting the best trees and for preserving genetic diversity. It is also recommended to establish permanent observation plots in oak populations in order to monitor the development and inheritance of quantitative traits that are controlled by multiple genes. A strategic plan for conservation of *Q. robur* populations (*in situ* and *ex situ*) will ensure the survival of the species under the ongoing climatic changes [265]. Recommendations for future *Q. robur* breeding and conservation programs should include the setup of experiments that will focus on genetic, phenological, chemical and morphometric analysis of *Q. robur* in natural conditions, greenhouses, and *in vitro* culture [266,267].

Finally, it is important to highlight that genomic selection shows significant promise for oaks, as it can match or even exceed the effectiveness of phenotypic selection for traits related to growth and wood quality. Genomic selection accelerates genetic improvement by increasing selection intensity, drastically reducing the generation interval, and enhancing the accuracy of estimated breeding values, thus boosting genetic gain per unit time [268,269,270].

In conclusion, traditional breeding and genomic selection of *Q. robur* should focus on selecting for both valuable timber traits and adaptation to climate change. Selection markers should target trunk morphology, wood properties, and the relationship between root and above-ground biomass. Plus stands, identified for their high-quality trees, are a key resource for breeding. These trees are selected for traits like growth rate, wood quality, and resistance to pests. Breeding and forestry efforts also prioritize the conservation of genetic diversity through *in situ* and *ex situ* programs. Genomic selection is increasingly used for accelerating improvements in growth and wood quality, offering advantages over traditional phenotypic selection methods.

## 14. Conclusions

The data of population genetics, genomics, phylogenetics, phylogeography, phenology, dendroecology, and dendrochronology presented in this review are important for determining the adaptive and evolutionary processes that contribute to the survival and distribution of *Q. robur* under a changing climate. We assume that the conservation and adaptation of *Q. robur* depends to a large extent on the accumulation of large reserves of genetic variability within populations, high-phenotypic plasticity, the ability to quickly migrate and introgressive hybridization, which contributes to adaptive introgression and facilitates migration. However, there are concerns regarding *Q. robur*’s adaptive response to current selection pressures. Some studies indicate that *Q. robur* may experience a gradual decline in certain regions, particularly in mixed stands with *Q. petraea*. This aligns with the differing demographic trends observed between the two species, suggesting that *Q. petraea* may have a competitive edge under certain environmental conditions [271]. However, fluctuations of selection and a rapid evolutionary response of oak populations to climatic transitions in the past suggest that similar trends may be at work now [272], and European oaks may survive and thrive in the changing environment with some adaptive management assistance.

## Figures and Tables

**Table 1:** Genome assembles of *Q. robur* available at the NCBI GenBank database.

Assembly (Type)	GenBank Accession Number	Level	Release	WGS Accession	Scaffold Count	Genome Size, Mbp	Submitter
ASM301314v1 (haploid)	GCA_003013145.1	Scaffold	Mar, 2018	PVWZ01	84,416	719.6	Swiss Institute of Bioinformatics
dhQueRobu3.1 (principal haplotype of diploid)	GCA_932294415.1 (NCBI RefSeq)	Chromosome	Mar, 2022	CAKOAN01	95	789.2	Wellcome Sanger Institute
dhQueRobu3.1 (alternate haplotype of diploid)	GCA_932294425.1	Scaffold	Mar, 2022	CAKOAP01	1219	762.4	Wellcome Sanger Institute
Q_robur_v1	GCA_900291515.1	Scaffold	Mar, 2018	OLKR01	550	814.3	Genoscope CEA

## References

[1] Bellusci G., Braglia R., Di Marco G., Redi E., Canini A., Gismondi A. (2023). Assessing molecular diversity among 87 species of the *Quercus* L. genus by RAPD markers. Genet. Resour. Crop Evol..

[2] Eaton E., Caudullo G., Oliveira S., de Rigo D., San-Miguel-Ayanz J., de Rigo D., Caudullo G., Houston Durrant T., Mauri A. (2016). Quercus robur and *Quercus petraea* in Europe: Distribution, habitat, usage and threats. European Atlas of Forest Tree Species.

[3] Mölder A., Meyer P., Nagel R.-V. (2019). Integrative management to sustain biodiversity and ecological continuity in Central European temperate oak (*Quercus robur*, *Q. petraea*) Forests: An Overview. For. Ecol. Manag..

[4] Mitchell R., Bellamy P., Ellis C., Hewison R., Hodgetts N., Iason G., Littlewood N., Newey S., Stockan J., Taylor A. (2019). OakEcol: A database of Oak-associated biodiversity within the UK. Data Brief.

[5] Johnson P.S., Shifley S.R., Rogers R. (2009). The Ecology and Silviculture of Oaks.

[6] Cañellas I., Sánchez-González M., Bogino S.M., Adame P., Moreno-Fernández D., Herrero C., Roig S., Tomé M., Paulo J.A., Bravo F., Bravo F., LeMay V., Jandl R. (2017). Carbon sequestration in Mediterranean oak forests. Managing Forest Ecosystems: The Challenge of Climate Change.

[7] Balaganesh B., Murali S., Sinduja D.K., Balamurugan V., Arunkumar R., Vignesh K., Suthin Raj T. (2022). Chapter 15—Carbon sequestration potential of oak tree. Compendium of Agriculture and Allied Sciences.

[8] Norby R.J., Loader N.J., Mayoral C., Ullah S., Curioni G., Smith A.R., Reay M.K., Van Wijngaarden K., Amjad M.S., Brettle D. (2024). Enhanced woody biomass production in a mature temperate forest under elevated CO_2_. Nat. Clim. Chang..

[9] Kovács B., Tinya F., Németh C., Ódor P. (2020). Unfolding the effects of different forestry treatments on microclimate in oak forests: Results of a 4-yr experiment. Ecol. Appl..

[10] Kovács B., Németh C., Aszalós R., Veres K. (2024). Small oases below the canopy: The cooling effects of water-filled tree holes on the local microclimate in oak-dominated stands. Agric. For. Meteorol..

[11] Verheyen K., Gillerot L., Blondeel H., De Frenne P., De Pauw K., Depauw L., Lorer E., Sanczuk P., Schreel J., Vanneste T. (2024). Forest canopies as nature-based solutions to mitigate global change effects on people and nature. J. Ecol..

[12] Zuazo V.H.D., Pleguezuelo C.R.R., Lichtfouse E., Navarrete M., Debaeke P., Véronique S., Alberola C. (2009). Soil-Erosion and Runoff Prevention by Plant Covers: A Review. Sustainable Agriculture.

[13] Rodrigues A.R., Botequim B., Tavares C., Pécurto P., Borges J.G. (2020). Addressing soil protection concerns in forest ecosystem management under climate change. For. Ecosyst..

[14] The Technical Properties of Oak Wood. https://hugokaempf.de/en/advisor/the-technical-properties-of-oak-wood.

[15] Santos J.A., Carvalho J.P.F., Santos J., Chuteira C.A., Grão A.B. (2012). Chapter 4—Oak Wood. Oak: Ecology, Types and Management.

[16] Muñoz G.R., Gete A.R. (2011). Relationships between mechanical properties of oak timber (*Q. robur* L.). Holzforschung.

[17] Jordão A.M., Cosme F. (2022). The Application of Wood Species in Enology: Chemical Wood Composition and Effect on Wine Quality. Appl. Sci..

[18] Pencák T., Dordevic D., Tremlová B. (2024). Utilization of Oak (genus) tree parts in food industry: A review. Maso Int.—J. Food Sci. Technol..

[19] Richard B., Bénard A., Dumarçay S., Colin F. (2024). Wood, knots and bark extractives for oak, beech and Douglas fir: A dataset based on a review of the scientific literature. Ann. For. Sci..

[20] Tomczak K., Mania P., Cukor J., Vacek Z., Komorowicz M., Tomczak A. (2024). Wood Quality of Pendulate Oak on Post-Agricultural Land: A Case Study Based on Physico-Mechanical and Anatomical Properties. Forests.

[21] Meyer L., Brischke C., Melcher E., Brandt K., Lenz M.T., Soetbeer A. (2014). Durability of English oak (*Quercus robur* L.)–Comparison of decay progress and resistance under various laboratory and field conditions. Int. Biodeterior. Biodegrad..

[22] Mills A. (2013). The British Oak.

[23] Acton J. (2024). Oaklore: Adventures in a World of Extraordinary Trees.

[24] Francis R., Dufraisse A. (2021). Firewood and timber collection and management strategies from early medieval sites in eastern England. Initial results from the anthraco-typological analysis of oak charcoal remains. Quat. Int..

[25] Malico I., Gonçalves A.C., Sousa A.M.O., Gonçalves A.C., Sousa A., Malico I. (2021). Evergreen oak biomass residues for firewood. Forest Biomass—From Trees to Energy.

[26] Jamali S., Haack R. (2024). From Glory to Decline: Uncovering Causes of Oak Decline in Iran. For. Pathol..

[27] Bhatia N., Friedman A., Del Rosso J. (2019). Applications of Topical Oak Bark Extract in Dermatology: Clinical Examples and Discussion. J. Drugs Dermatol..

[28] Şöhretoğlu D., Renda G. (2020). The polyphenolic profile of Oak (*Quercus*) species: A phytochemical and pharmacological overview. Phytochem. Rev..

[29] Häsler Gunnarsdottir S., Sommerauer L., Schnabel T., Oostingh G.J., Schuster A. (2023). Antioxidative and Antimicrobial Evaluation of Bark Extracts from Common European Trees in Light of Dermal Applications. Antibiotics.

[30] Logan W.B. (2006). Oak: The Frame of Civilization.

[31] Leroy T., Plomion C., Kremer A. (2020). Oak Symbolism in the Light of Genomics. New Phytol..

[32] Elam K. (2022). Romancing the Oak: On the Performativity of Trees in Shakespearean Comedy. Fascinating Rhythms.

[33] Sabouri S., Javadi S. (2022). Oak; A Heritage, a Culture. J. Art Civiliz. Orient.

[34] Oosterbaan A., Leffef F. (1987). Decline in health and death of *Quercus robur* L. in the Netherlands. Nederlands-Bosbouwtijdschrift.

[35] Thomas F.M., Blank R., Hartmann G. (2002). Abiotic and biotic factors and their interactions as causes of oak decline in Central Europe. For. Pathol..

[36] Neagu S. (2010). Long-term growth decline of *Quercus robur* L. forests in Vlăsia Plain. Proc. Rom. Acad. Ser. B Chem. Life Sci. Geosci..

[37] Lione G.G., Ebone A., Petrella F., Terzuolo P., Nicolotti G., Gonthier P. (2012). Decline of *Quercus Robur* Forests in Northwestern Italy: Current Situation and Tentative Aetiology. IOBC/Wprs Bull..

[38] Matisons R., Elferts D., Brūmelis G. (2013). Possible signs of growth decline of pedunculate oak in Latvia during 1980–2009 in treering width and vessel size. Bal. For..

[39] Keča N., Koufakis I., Dietershagen J., Nowakowska J.A., Oszako T. (2016). European Oak Decline Phenomenon in Relation to Climatic Changes. Folia For. Pol. Ser. A.

[40] Conte A.L., Di Pietro R., Iamonico D., Di Marzio P., Cillis G., Lucia D., Fortini P. (2019). Oak decline in the Mediterranean Basin: A Study Case from the Southern Apennines (Italy). Plant Sociol..

[41] Losseau J., Jonard M., Vincke C. (2020). Pedunculate oak decline in southern Belgium: A long-term process highlighting the complex interplay among drought, winter frost, biotic attacks, and masting. Can. J. For. Res..

[42] Schroeder H., Nosenko T., Ghirardo A., Fladung M., Schnitzler J.P., Kersten B. (2021). Oaks as Beacons of Hope for Threatened Mixed Forests in Central Europe. Front. For. Glob. Chang..

[43] Macháčová M., Nakládal O., Samek M., Baťa D., Zumr V., Pešková V. (2022). Oak Decline Caused by Biotic and Abiotic Factors in Central Europe: A Case Study from the Czech Republic. Forests.

[44] Kowsari M., Karimi E. (2023). A review on oak decline: The global situation causative factors and new research approaches. For. Syst..

[45] Gribbe S., Enderle L., Weigel R., Hertel D., Leuschner C., Muffler L. (2024). Recent growth decline and shifts in climatic growth constraints suggest climate vulnerability of beech, Douglas fir, pine and oak in Northern Germany. For. Ecol. Manag..

[46] Krutovsky K.V. (2022). Dendrogenomics Is a New Interdisciplinary Field of Research of the Adaptive Genetic Potential of Forest Tree Populations Integrating Dendrochronology, Dendroecology, Dendroclimatology, and Genomics. Russ. J. Genet..

[47] Gailing O., Hipp A.L., Plomion C., Carlson J.E., Rajora O.P. (2021). Oak Population Genomics. Population Genomics: Forest Trees.

[48] Gadella T.W.J., Kliphuis E. (1973). Chromosome numbers of flowering plants in the Netherlands. VI. Meded. Het Bot. Mus. Herb. Rijksuniv. Utrecht.

[49] Butorina A.K. (1993). Cytogenetic study of diploid and spontaneous triploid oaks, *Quercus robur* L.. Ann. For. Sci..

[50] Naujoks G., Hertel H., Ewald D. (1995). Characterisation and propagation of an adult triploid pedunculate oak (*Quercus robur*). Silvae Genet..

[51] Dzialuk A., Chybicki I., Welc M., Sliwinska E., Burczyk J. (2007). Presence of Triploids among Oak Species. Ann. Bot..

[52] Zoldoš V., Besendorfer V., Jelenić S., Lorković Z., Littvay T., Papeš D., Borzan Ž., Schlarbaum S.E. (1997). Cytogenetic damages as an indicator of pedunculate oak forest decline. Proceedings of the First IUFRO Cytogenetics Working Party S2.04-08 Symposium Cytogenetic Studies of Forest Trees and Shrub Species.

[53] Zoldos V., Papes D., Brown S., Panaud O., Siljak-Yakovlev S. (1998). Genome size and base composition of seven *Quercus* species: Inter- and intra-population variation. Genome.

[54] Mehra P.N., Hans A.S., Sareen T.S. (1972). Cytomorphology of Himalayan Fagaceae. Silvae Genet..

[55] D’emerico S., Bianco P., Medagli P., Schirone B. (1995). Karyotype analysis in *Quercus* spp. (Fagaceae). Silvae Genet..

[56] Ohri D., Ahuja M.R. (1990). Giemsa C-banding in *Quercus* L. (oak). Silvae Genet..

[57] Ohri D., Ahuja M.R. (1991). Giemsa C-banding in *Fagus sylvatica* L., *Betula pendula* Roth, and *Populus tremula* L.. Silvae Genet..

[58] Wang L.-m. (1986). A taxonomy study of the deciduous Oak in China by means of cluster and karyotype analysis. Bull. Bot. Res..

[59] Cao R.B., Chen R., Liao K.X., Li H., Xu G.B., Jiang X.L. (2024). Karyotype and LTR-RTs analysis provide insights into oak genomic evolution. BMC Genom..

[60] Natividade J.V. (1937). Recherches cytologiques sur quelque especes et hybrides du genre *Quercus* (I). Bol. Sociedate Broteriana.

[61] Leitch I.J., Johnston E., Pellicer J., Hidalgo O., Bennett M.D. (2019). Angiosperm DNA C-Values Database (Release 9.0, April 2019). https://cvalues.science.kew.org/search/angiosperm.

[62] Favre J.M., Brown S. (1996). A flow cytometric evaluation of the nuclear DNA content and GC percent in genomes of European oak species. Ann. Sci. For..

[63] Kremer A., Casasoli M., Barreneche T., Bodénès C., Sisco P., Kubisiak T., Scalfi M., Leonardi S., Bakker E., Buiteveld J., Kole C. (2007). Fagaceae Trees. Genome Mapping and Molecular Breeding in Plants.

[64] Schwarz O. (1964). *Quercus* L. In Flora Europaea. Lycopodiaceae Platanaceae.

[65] Camus A. (1938). Les chênes, Monographie du genre *Quercus* et Monographie du genre Lithocarpus. Encyclopédie Economique de Sylviculture.

[66] Manos P.S., Steele K.P. (1997). Phylogenetic analyses of “higher” Hamamelidiae based on plastid sequence data. Am. J. Bot..

[67] Xu L.A. (2004). Diversité de l’ADN Chloroplastique et Relations Phylogénétiques au Sein des Fagacées et du Genre *Quercus*. Ph.D. Thesis.

[68] Barreneche T., Bodénès C., Lexer C., Trontin J.F., Fluch S., Streiff R., Plomion C., Roussel G., Steinkellner H., Burg K. (1998). A genetic linkage map of *Quercus robur* L. (pedunculate oak) based on RAPD, SCAR, microsatellite, minisatellite, isozyme and 5S rDNA markers. Theor. Appl. Genet..

[69] Barreneche T., Casasoli M., Russell K., Akkak A., Meddour H., Plomion C., Villani F., Kremer A. (2004). Comparative mapping between *Quercus* and *Castanea* using simple-sequence repeats (SSRs). Theor. Appl. Genet..

[70] Gailing O. (2008). QTL analysis of leaf morphological characters in a *Quercus robur* full-sib family (*Q. robur* × *Q. robur* ssp. *slavonica*). Plant Biol..

[71] Gailing O., Langenfeld-Heyser R., Polle A., Finkeldey R. (2008). Quantitative trait loci affecting stomatal density and growth in a *Quercus robur* progeny: Implications for the adaptation to changing environments. Glob. Chang. Biol..

[72] Bodénès C., Chancerel E., Gailing O., Vendramin G.G., Bagnoli F., Durand J., Goicoechea P.G., Soliani C., Villani F., Mattioni C. (2012). Comparative mapping in the Fagaceae and beyond with EST-SSRs. BMC Plant Biol..

[73] Bodénès C., Chancerel E., Ehrenmann F., Kremer A., Plomion C. (2016). High-density linkage mapping and distribution of segregation distortion regions in the oak genome. DNA Res..

[74] Quercus PORTAL A European Genetic and Genomic Web Resources for Quercus: Oak Mapping Initiatives. https://quercusportal.pierroton.inra.fr/index.php?p=GENETIC_MAPPING.

[75] Lepoittevin C., Bodénès C., Chancerel E., Villate L., Lang T., Lesur I., Boury C., Ehrenmann F., Zelenica D., Boland A. (2015). Single-nucleotide polymorphism discovery and validation in high-density SNP array for genetic analysis in European white oaks. Mol. Ecol. Resour..

[76] Endelman J., Plomion C. (2014). LPmerge: An R package for merging genetic maps by linear programming. BioInformatics.

[77] Plomion C., Aury J.M., Amselem J., Alaeitabar T., Barbe V., Belser C., Bergès H., Bodénès C., Boudet N., Boury C. (2016). Decoding the oak genome: Public release of sequence data, assembly, annotation and publication strategies. Mol. Ecol. Resour..

[78] Plomion C., Aury J., Amselem J., Leroy T., Murat F., Duplessis S., Faye S., Francillonne N., Labadie K., Provost G.L. (2018). Oak genome reveals facets of long lifespan. Nat. Plants.

[79] Oak Genome Browser. https://urgi.versailles.inra.fr/WebApollo_oak_PM1N/PseudoMolecule.html.

[80] Dumolin S., Demesure B., Petit R.J. (1995). Inheritance of chloroplast and mitochondrial genomes in pedunculate oak investigated with an efficient PCR method. Theoret. Appl. Genet..

[81] Semerikova S.A., Isakov I.Y., Semerikov V.L. (2021). Chloroplast DNA Variation and Phylogeography of Pedunculate Oak *Quercus robur* L. in the Eastern Part of the Range. Russ. J. Genet..

[82] Ducousso A., Bordacs S. (2004). EUFORGEN Technical Guidelines for Genetic Conservation and Use for Pedunulate and Sessile Oaks (Quercus robur and Q. petraea).

[83] Cottrell J.E., Munro R.C., Tabbener H.E., Gillies A.C.M., Forrest G.I., Deans J.D., Lowe A.J. (2002). Distribution of chloroplast variation in British oaks (*Quercus robur* and *Q. petraea*): The influence of postglacial recolonization and human management. For. Ecol. Manag..

[84] Petit R.J., Kremer A., Wagner D.B. (1993). Geographic structure of chloroplast DNA polymorphisms in European oaks. Theor. Appl. Genet..

[85] Petit R.J., Vendramin G.G., Weiss S., Ferrand N. (2007). Plant phylogeography based on organelle genes: An introduction. Phylogeography of Southern European Refugia.

[86] Petit R.J., Brewer S., Bordács S., Burg K., Cheddadi R., Coart E., Coart E., Cottrell J., Csaikl U.M., van Damh B. (2002). Identification of refugia and post-glacial colonisation routes of European white oaks based on chloroplast DNA and fossil pollen evidence. For. Ecol. Manag..

[87] Petit R.J., Csaikl U.M., Bordacs S., Burg K., Coart E., Cottrell J., van Dam B., Deans J.D., Dumolin-Lapègue S., Fineschi S. (2002). Chloroplast DNA variation in European white oaks—Phylogeography and patterns of diversity based on data from over 2600 populations. For. Ecol. Manag..

[88] Blanc-Jolivet C., Liesebach M. (2015). Tracing the origin and species identity of *Quercus robur* and *Quercus petraea* in Europe: A review. Silvae Genet..

[89] Lazic D., Hipp A.L., Carlson J.E., Gailing O. (2021). Use of Genomic Resources to Assess Adaptive Divergence and Introgression in Oaks. Forests.

[90] Gailing O., Wachter H., Heyder J., Rogge M., Finkeldey R. (2009). Chloroplast DNA analyses of very old, presumably autochthonous *Quercus robur* L. stands in North Rhine-Westphalia. Allg. Forst-u. Jagdztg..

[91] Bi Q., Li D., Zhao Y., Wang M., Li Y., Liu X., Wang L., Yu H. (2019). Complete mitochondrial genome of *Quercus variabilis* (Fagales, Fagaceae). Mitochondrial DNA B Resour..

[92] Wang L., Li L.-L., Chen L., Zhang R.-G., Zhao S.-W., Yan H., Gao J., Chen X., Si Y.-J., Chen Z. (2023). Telomere-to-telomere and haplotype-resolved genome assembly of the Chinese cork oak (*Quercus variabilis*). Front. Plant Sci..

[93] Liu D., Guo H., Zhu J., Qu K., Chen Y., Guo Y., Ding P., Yang H., Xu T., Jing Q. (2022). Complex Physical Structure of Complete Mitochondrial Genome of *Quercus acutissima* (Fagaceae): A Significant Energy Plant. Genes.

[94] Usié A., Serra O., Barros P.M., Barbosa P., Leão C., Capote T., Almeida T., Rodrigues L., Carrasquinho I., Guimarães J.B. (2023). An improved reference genome and first organelle genomes of *Quercus suber*. Tree Genet. Genomes.

[95] Grosser M.R., Sites S.K., Murata M.M., Lopez Y., Chamusco K.C., Love Harriage K., Grosser J.W., Graham J.H., Gmitter F., Chase C.D. (2023). Plant mitochondrial introns as genetic markers-conservation and variation. Front. Plant Sci..

[96] Mosca E., Cruz F., Gómez-Garrido J., Bianco L., Rellstab C., Brodbeck S., Csilléry K., Fady B., Fladung M., Fussi B. (2019). A reference genome sequence for the European silver fir (*Abies alba* Mill.): A community-generated genomic resource. G3 Genes Genomes Genet..

[97] Putintseva Y.A., Bondar E.I., Simonov E.P., Sharov V.V., Oreshkova N.V., Kuzmin D.A., Konstantinov Y.M., Shmakov V.N., Belkov V.I., Sadovsky M.G. (2020). Siberian larch (*Larix sibirica* Ledeb.) mitochondrial genome assembled using both short and long nucleotide sequence reads is currently the largest known mitogenome. BMC Genom..

[98] Feng L., Wang Z., Wang C., Yang X., An M., Yin Y. (2023). Multichromosomal mitochondrial genome of *Punica granatum*: Comparative evolutionary analysis and gene transformation from chloroplast genomes. BMC Plant Biol..

[99] Wang Y., Cui G., He K., Xu K., Liu W., Wang Y., Wang Z., Liu S., Bi C. (2024). Assembly and Comparative Analysis of the Complete Mitochondrial Genome of *Ilex rotunda* Thunb. Forests.

[100] Wu C.S., Wang R.J., Chaw S.M. (2024). Integration of large and diverse angiosperm DNA fragments into Asian *Gnetum* mitogenomes. BMC Biol..

[101] Mullagulov R.Y., Redkina N.N., Yanbaev Y.A. (2008). Allozyme variability of pedunculate oak *Quercus robur* L. (Fagaceae) in isolated populations on the eastern border of the range. Bull. Orenbg. State Univ..

[102] Popović M., Katičić Bogdan I., Varga F., Šatović Z., Bogdan S., Ivanković M. (2023). Genetic Diversity in Peripheral Pedunculate Oak (*Quercus robur* L.) Provenances—Potential Climate Change Mitigators in the Center of Distribution despite Challenges in Natural Populations. Forests.

[103] Semerikova S.A., Tashev A.N., Semerikov V.L. (2023). Genetic Diversity and History of Pedunculate Oak *Quercus robur* L. in the East of the Range. Russ. J. Ecol..

[104] Degen B., Yanbaev Y., Ianbaev R., Blanc-Jolivet C., Mader M., Bakhtina S. (2022). Large-scale genetic structure of *Quercus robur* in its eastern distribution range enables assignment of unknown seed sources. Forestry.

[105] Gömöry D., Yakovlev I., Zhelev P., Jedináková J., Paule L. (2001). Geneticd ifferentiation of oak populations within the *Quercus robur*/*Quercus petraea* complex in Central and Eastern Europe. Heredity.

[106] Degen B., Yanbaev Y., Mader M., Ianbaev R., Bakhtina S., Schroeder H., Blanc-Jolivet C. (2021). Impact of Gene Flow and Introgression on the Range Wide Genetic Structure of *Quercus robur* (L.) in Europe. Forests.

[107] Yakovlev I.A., Kleinschmidt J. (2002). Genetic differentiation of pedunculate oak *Quercus robur* L. in the European part of Russia based on RAPD markers. Russ. J. Genet..

[108] Gabitova A.A., Yanbaev R.Y., Redkina N.N. (2015). High genetic polymorphism in a population of the English oak in the lowlands of Western Ural’s macro-slope. Vestn. Bashk. Univ. Ser. Biol..

[109] Chokheli V., Kagan D.I., Varduny T.V., Kozlovsky B., Sereda M., Kapralova O.A., Dmitriev P., Padutov V.E. (2018). Ecological and genetic differentiation of populations of *Quercus robur* L. in the Rostov Region with the use of ISSR-markers. Turczaninowia.

[110] Ballian D., Belletti P., Ferrazzini D., Bogunić F., Kajba D. (2010). Genetic variability of pedunculate oak (*Quercus robur* L.) in Bosnia and Herzegovina. Period. Biol..

[111] Degen B., Yanbaev Y., Ianbaev R., Bakhtina S., Tagirova A. (2021). Genetic diversity and differentiation among populations of the pedunculate oak (*Quercus robur*) at the eastern margin of its range based on a new set of 95 SNP loci. J. For. Res..

[112] Avanzi C., Bagnoli F., Romiti E., Spanu I., Tsuda Y., Vajana E., Vendramin G.G., Piotti A. (2023). The latitudinal trend in genetic diversity and distinctiveness of *Quercus robur* rear edge forest remnants calls for new conservation priorities. bioRxiv.

[113] Di Pietro R., Quaranta L., Mattioni C., Simeone M.C., Di Marzio P., Proietti E., Fortini P. (2024). Chloroplast Haplotype Diversity in the White Oak Populations of the Italian Peninsula, Sicily, and Sardinia. Forests.

[114] Neophytou C., Aravanopoulos F.A., Fink S., Dounavi A. (2010). Detecting interspecific and geographic differentiation patterns in two interfertile oak species (*Quercus petraea* (Matt.) Liebl. and *Q. robur* L.) using small sets of microsatellite markers. For. Ecol. Manag..

[115] Milesi P., Kastally C., Dauphin B., Cervantes S., Bagnoli F., Budde K.B., Cavers S., Fady B., Faivre-Rampant P., González-Martínez S.C. (2024). Resilience of genetic diversity in forest trees over the Quaternary. Nat. Commun..

[116] Kremer A., Delcamp A., Lesur I., Wagner S., Rellstab C., Guichoux E., Leroy T. (2024). Whole-genome screening for near-diagnostic genetic markers for four western European white oak species identification. Ann. For. Sci..

[117] Jurkšienė G., Baranov O.Y., Kagan D.I., Kovalevič-Razumova O.A., Baliuckas V. (2020). Genetic diversity and differentiation of pedunculate (*Quercus robur*) and sessile (*Q. petraea*) oaks. J. For. Res..

[118] Leroy T., Louvet J.-M., Lalanne C., Le Provost G., Labadie K., Aury J.-M., Delzon S., Plomion C., Kremer A. (2020). Adaptive introgression as a driver of local adaptation to climate in European white oaks. New Phytol..

[119] Leroy T., Rougemont Q., Dupouey J.-L., Bodénès C., Lalanne C., Belser C., Labadie K., Le Provost G., Aury J.-M., Kremer A. (2020). Massive postglacial gene flow between European white oaks uncovered genes underlying species barriers. New Phytol..

[120] Cannon C.H., Petit R.J. (2020). The oak syngameon: More than the sum of its parts. New Phytol..

[121] Karunarathne P., Zhou Q., Lascoux M., Milesi P. (2024). Hybridization mediated range expansion and climate change resilience in two keystone tree species of boreal forests. Glob. Chang. Biol..

[122] Zhou Q., Karunarathne P., Andersson-Li L., Chen C., Opgenoorth L., Heer K., Piotti A., Vendramin G.G., Nakvasina E., Lascoux M. (2024). Recurrent hybridization and gene flow shaped Norway and *Siberian spruce* evolutionary history over multiple glacial cycles. Mol. Ecol..

[123] Leroy T., Roux C., Villate L., Bodénès C., Romiguier J., Paiva J.A.P., Dossat C., Aury J.-M., Plomion C., Kremer A. (2017). Extensive recent secondary contacts between four European white oak species. New Phytol..

[124] Fu R., Zhu Y., Liu Y., Feng Y., Lu R., Li Y., Li P., Kremer A., Lascoux M., Chen J. (2022). Genome-wide analyses of introgression between two sympatric Asian oak species. Nat. Ecol. Evol..

[125] Correia B., Valledor L., Meijón M., Rodriguez J.L., Dias M.C., Santos C., Cañal M.J., Rodriguez R., Pinto G. (2013). Is the interplay between epigenetic markers related to the acclimation of cork oak plants to high temperatures?. PLoS ONE.

[126] Sork V.L., Browne L., Fitz-Gibbon S., Pellegrini M. (2019). Potential Role of Epigenetic Processes in Oak Populations. Int. Oaks.

[127] Escandón M., Castillejo M.Á., Jorrín-Novo J.V., Rey M.-D. (2021). Molecular Research on Stress Responses in *Quercus* spp.: From Classical Biochemistry to Systems Biology through Omics Analysis. Forests.

[128] Silva H.G., Sobral R.S., Magalhães A.P., Morais-Cecílio L., Costa M.M.R. (2020). Genome-Wide Identification of Epigenetic Regulators in *Quercus suber* L.. Int. J. Mol. Sci..

[129] Labella-Ortega M. (2024). Genetic and Epigenetic Bases of *Quercus ilex* Variability. Ph.D. Thesis.

[130] Lesur I., Rogier O., Sow M.D., Boury C., Duplan A., Garnier A., Senhaji-Rachik A., Civan P., Daron J., Delaunay A. (2024). A strategy for studying epigenetic diversity in natural populations: Proof of concept in poplar and oak. J. Exp. Bot..

[131] Gugger P.F., Fitz-Gibbon S.T., Pellegrini M., Sork V.L. (2016). Species-wide patterns of DNA methylation variation in *Quercus lobata* and their association with climate gradients. Mol. Ecol..

[132] Platt A., Gugger P.F., Pellegrini M., Sork V.L. (2015). Genome-wide signature of local adaptation linked to variable CpG methylation in oak populations. Mol. Ecol..

[133] Fuchs J., Jovtchev G., Schubert I. (2008). The chromosomal distribution of histone methylation marks in gymnosperms differs from that of angiosperms. Chromosome Res..

[134] Vičić V., Barišić D., Horvat T., Biruš I., Zoldos V. (2013). Epigenetic characterization of chromatin in cycling cells of pedunculate oak, *Quercus robur* L.. Tree Genet. Genomes.

[135] Rubio B., Provost G.L., Brachi B., Gerardin T., Brendel O., Tost J., Daviaud C., Gallusci P. (2023). Species-specific epigenetic responses to drought stress of two sympatric oak species reflect their ecological preferences. bioRxiv.

[136] Ramírez-Valiente J.A., Sánchez-Gómez D., Aranda I., Valladares F. (2010). Phenotypic Plasticity and Local Adaptation in Leaf Ecophysiological Traits of 13 Contrasting Cork Oak Populations under Different Water Availabilities. Tree Physiol..

[137] Mátyás C. (2021). Adaptive pattern of phenotypic plasticity and inherent growth reveal the potential for assisted transfer in sessile oak (*Quercus petraea* L.). For. Ecol. Manag..

[138] Solé-Medina A., Robledo-Arnuncio J.J., Ramírez-Valiente J.A. (2022). Multi-trait genetic variation in resource-use strategies and phenotypic plasticity correlates with local climate across the range of a Mediterranean oak (*Quercus faginea*). New Phytol..

[139] Kormann J.M., van der Maaten-Theunissen M., Unterholzner L., Liesebach M., Liepe K.J., van der Maaten E. (2024). Variation in vessel traits of northern red oak (*Quercus rubra* L.) provenances revealed high phenotypic plasticity to prevailing environmental conditions. Trees.

[140] Lesur I., Le Provost G., Bento P., Da Silva C., Leplé J.-C., Murat F., Ueno S., Bartholomé J., Lalanne C., Ehrenmann F. (2015). The oak gene expression atlas: Insights into Fagaceae genome evolution and the discovery of genes regulated during bud dormancy release. BMC Genom..

[141] Derory J., Léger P., Garcia V., Schaeffer J., Hauser M.T., Salin F., Luschnig C., Plomion C., Glössl J., Kremer A. (2006). Transcriptome analysis of bud burst in sessile oak (*Quercus petraea*). New Phytol..

[142] Kóscielniak P., Glazínska P., Zadworny M. (2022). OakRootRNADB—A consolidated RNA-seq database for coding and noncoding RNA in roots of pedunculate oak (*Quercus robur*). Database.

[143] Baliuckas V., Pliura A. (2003). Genetic variation and phenotypic plasticity of *Quercus robur* populations and open-pollinated families in Lithuania. Scand. J. For. Res..

[144] Hautsalo J., Mathieu P., Elshibli S., Vakkari P., Raisio J., Pulkkinen P. (2015). Variation in height and survival among northern populations of pedunculate oak (*Quercus robur* L.): Results of a 13-year field study. Silva Fenn..

[145] George J.P., Theroux-Rancourt G., Rungwattana K., Scheffknecht S., Momirovic N., Neuhauser L., Weißenbacher L., Watzinger A., Hietz P. (2020). Assessing adaptive and plastic responses in growth and functional traits in a 10-year-old common garden experiment with pedunculate oak (*Quercus robur* L.) suggests that directional selection can drive climatic adaptation. Evol. Appl..

[146] Kaplina N.F. (2019). Influence of Crown Development on Radial Increment of Early and Late Stem Wood of *Quercus robur*. Vestn. Povolzhskogo Gos. Tekhnol. Univ. Seriya Les. Ekologiya. Prir. (Vestn. Volga State Univ. Technol. Ser. For. Ecol. Nat. Manag.).

[147] Semerikov L.F., Glotov N.V. (1983). Population structure of pedunculate oak. Physiological and Population Variability.

[148] Gneusheva T.M., Kozhevnikov A.P., Krutov A.P. (2012). Intraspecific differentiationof English oak (*Quercus robur* L.) into intrapopulation groups, geographical and ecological populations in different parts of the area. Sci. News Belgorod State Univ. Ser. Nat. Sci..

[149] Kryukova S.A., Shirnin V.K. (2016). Fruiting of oak forests and plus-sized oak trees. Lesotekhnicheskii Zhurnal (For. Eng. J.).

[150] Storozhenko V.G., Chebotarev P.A., Chebotareva V.V., Zasadnaya V.A. (2020). Wood Biomass Stock Allocated to the Main Forest-Forming Species of the Southern Forest Steppes’ Stands. Lesovedenie.

[151] Chernodubov A.I. (2018). Variability of acorns of the Voronezh upland oak grove. Actual Dir. Sci. Res. XXI Century Theory Pract..

[152] McClory R., Ellis R.H., Lukac M., Clark J. (2024). Pollen source affects acorn production in pedunculate oak (*Quercus robur* L.). J. For. Res..

[153] Batos B. (2012). Diversity of Pedunculate Oak (Quercus robur L.).

[154] Batos B., Miljković D. (2017). Pollen viability in *Quercus robur* L.. Arch. Biol. Sci..

[155] Batos B., Miljković D., Ninić-Todorović J. (2012). Length of vegetation period as parameter of common oak (*Quercus robur* L.) phenological variability. Genetika.

[156] Bobinac M., Batos B., Miljković D., Radulović S. (2012). Polycyclism and phenological variability in the common oak (*Quercus robur* L.). Arch. Biol. Sci..

[157] Šušić N.M., Bobinac M., Kerkez I., Živković A.B., Vojinović N. (2016). Height growth characteristics of one-year-old northern red oak seedlings (*Quercus rubra* L.) in full light conditions. Reforesta.

[158] Silchenko I.I. (2012). Phenological forms of pedunculate oak (*Quercus robur* L.) in various types of landscapes of the Bryansk region. Bull. Bryansk State Univ..

[159] Danilov M.D., Guryev V.D., Fedorov P.N., Mamaev S.A., Makhnev A.K. (1975). Some features of the population structure of pedunculate oak under conditions of the northeastern part of its range. Regularities of Intraspecific Variability of Deciduous Tree Species.

[160] Efimov Y.P. (1975). On the question of the territorial distribution of the phenological forms of the petiolate oak. Central Research Institute of Forest Genetics and Breeding (ЦНИИЛГиС) “Genetics, Selection, and Introduction of Forest Species”.

[161] En’kova E.I. (1976). Tellermanovskii Les i ego Vosstanovlenie (Tellerman Forest and Its Recovery).

[162] Puchałka R., Koprowski M., Przybylak R. Fenologia liści i ksylogeneza w zróżnicowanej wiekowo populacji dębu szypułkowego. Klimatyczne uwarunkowania życia lasu. Proceedings of the Ogólnopolska Konferencja Naukowa, Streszczenia Referatów.

[163] Slepykh O.O. (2016). Rhythm of phenology and distribution phenological forms of Pedunculate oak (*Quercus robur* L.) in Donetsk region. Biol. Syst..

[164] Wesołowski T., Rowiński P. (2008). Late leaf development in pedunculate oak (*Quercus robur*): An antiherbivore defence?. Scand. J. For. Res..

[165] Orlović S., Šimunovački D., Djorđević Z., Pilipović A., Radosavljević N. (2008). Očuvanje Genofonda I Proizvodnja Semena Hrasta Lužnjaka (*Quercus robur* L.). 250 Godina Ravnog Srema.

[166] Utkina I., Rubtsov V. (2017). Studies of Phenological Forms of Pedunculate Oak. Contemp. Probl. Ecol..

[167] Pirko Y.V., Netsvetov M., Kalafat L.O., Pirko N.M., Rabokon A.M., Privalikhin S.M., Demkovich A.Y., Bilonozhko Y.O., Blume Y.B. (2018). Genetic features of the phenological forms of *Quercus robur* (Fagaceae) according to the analysis of the introns polymorphism of β-tubulin genes and microsatellite loci. Ukr. Bot. J..

[168] Ueno S., Klopp C., Noirot C., Léger V., Prince E., Kremer A., Plomion C., Le Provost G. (2011). Detection of genes involved in bud phenology in sessile oak (*Quercus petraea* Matt. Liebl) combining digital expression analysis and Q-PCR. BMC Proc..

[169] Batos B., Šešlija Jovanović D., Miljković D. (2014). Spatial and temporal variability of flowering in the pedunculate oak (*Quercus robur* L.). Šumarski List..

[170] Andrić I., Jazbec A., Pintar V., Kajba D. (2018). Modelling the timing of leaf unfolding in pedunculate oak (*Quercus robur* L.) clonal seed orchard. Šumarski List.

[171] Tikvić I., Seletković Z., Ugarković D. (2006). The relationship between phenoform development of pedunculate oak and forest soil microclimate. Glas. Šumske Pokuse.

[172] Chokheli V., Kozlovsky B., Sereda M., Lysenko V., Fesenko I., Varduny T., Kapralova O., Bondarenko E. (2016). Preliminary comparative analysis of phenological varieties of *Quercus robur* by ISSR-markers. J. Bot..

[173] Puchałka R., Koprowski M., Przybylak J., Przybylak R., Dąbrowski H.P. (2016). Did the late spring frost in 2007 and 2011 affect tree-ring width and earlywood vessel size in Pedunculate oak (*Quercus robur*) in northern Poland?. Int. J. Biometeorol..

[174] Puchałka R., Koprowski M., Gričar J., Przybylak R. (2017). Does tree-ring formation follow leaf phenology in Pedunculate oak (*Quercus robur* L.)?. Eur. J. For. Res..

[175] Kitin P. (1992). Dynamics of cambial activity in the stem of early- and late-flushing forms of oak (*Quercus robur* vars. *praecox* and *tardiflora*) in the Park of Freedom, Sofia. Nauk. Gorata.

[176] Kostić S., Orlović S., Karaklić V., Kesić L., Zorić M., Stojanović D.B. (2021). Allometry and Post-Drought Growth Resilience of Pedunculate Oak (*Quercus robur* L.) Varieties. Forests.

[177] Koval I.M., Kostyashkin D.C. (2015). The influence of climate and recreation on formation of layers of annual wood of early and late forms *Quercus robur* L. in Kharkiv. Greenbelt. Sci. Bull. UNFU.

[178] Levlev V.V. (1970). Ecotypes and Forms of the Petiolate Oak in the Voronezh Nature Reserve: Abstract of the Dissertation of the Phd of Agricultural Sciences.

[179] Lukyanets V.B. (1979). Intraspecific Variability of the Petiolate Oak in the Central Forest-Steppe.

[180] Shitov V.P. (1986). The Form Diversity of Floodplain Oak Forests of Polesie and the Ways of Their Economic Use: Abstract of the Dissertation of the Phd of Agricultural Sciences.

[181] Shutyaev A.M. (1998). Biodiversity of the Pedunculate oak (Quercus robur L.) and Its Use in Breeding and Afforestation: Abstract of the Dissertation of the Dr. Agricultural Sciences.

[182] Kobranov N.P. (1925). Oak Breeding.

[183] Pukacka S., Bugała W. (2006). Wzrost i rozwój. Dęby. Quercus robur L., Quercus petraea Liebl.

[184] Antin C., Pélissier R., Vincent G., Couteron P. (2013). Crown allometries are less responsive than stem allometry to tree size and habitat variations in an Indian monsoon forest. Trees Struct. Funct..

[185] Rubtsov V.V., Utkina I.A. (2008). Adaptatsionnyye Reaktsii Duba na Defoliatsiyu.

[186] Vikhrov V.E. (1954). Stroenie i Fiziko-Mekhanicheskie Svoistva Drevesiny duba.

[187] van Asch M., Visser M.E. (2007). Phenology of forest caterpillars and their host trees: The importance of synchrony. Ann. Rev. Entomol..

[188] Wagenhoff E., Blum R., Engel K., Veit H., Delb H. (2013). Temporal synchrony of *Thaumetopoea processionea* egg hatch and *Quercus robur* budburst. J. Pest Sci..

[189] Milenin A.I., Popova A.A., Shestibratov K.A. (2023). Effect of Type of Forest Growth Conditions and Climate Elements on the Dynamics of Radial Growth in English Oak (*Quercus robur* L.) of Early and Late Phenological Forms. Forests.

[190] Denk T., Grimm G.W., Manos P.S., Deng M., Hipp A.L., Gil-Pelegrín E., Peguero-Pina J., Sancho-Knapik D. (2017). An Updated Infrageneric Classification of the Oaks: Review of Previous Taxonomic Schemes and Synthesis of Evolutionary Patterns. Oaks Physiological Ecology. Exploring the Functional Diversity of Genus Quercus L..

[191] Hipp A.L., Manos P.S., González-Rodríguez A., Hahn M., Kaproth M., McVay J.D., Avalos S.V., Cavender-Bares J. (2018). Sympatric Parallel Diversification of Major Oak Clades in the Americas and the Origins of Mexican Species Diversity. New Phytol..

[192] Hipp A.L., Manos P.S., Hahn M., Avishai M., Bodénès C., Cavender-Bares J., Crowl A.A., Deng M., Denk T., Fitz-Gibbon S. (2020). Genomic Landscape of the Global Oak Phylogeny. New Phytol..

[193] Kremer A., Abbott A.G., Carlson J.E., Manos P.S., Plomion C., Sisco P., Staton M.E., Ueno S., Vendramin G.G. (2012). Genomics of Fagaceae. Tree Genet. Genomes.

[194] Hipp A.L. (2024). Oak Origins: From Acorns to Species and the Tree of Life.

[195] Kremer A., Hipp A.L. (2020). Oaks: An evolutionary success story. New Phytol..

[196] Gosling R.H., Jackson R.W., Elliot M., Nichols C.P. (2024). Oak declines: Reviewing the evidence for causes, management implications and research gaps. Ecol. Solut. Evid..

[197] Sever K., Bogdan S., Škvorc Ž. (2023). Response of photosynthesis, growth, and acorn mass of pedunculate oak to different levels of nitrogen in wet and dry growing seasons. J. For. Res..

[198] Kebert M., Kostić S., Stojnić S., Čapelja E., Markić A.G., Zorić M., Kesić L., Flors V. (2023). A Fine-Tuning of the Plant Hormones, Polyamines and Osmolytes by Ectomycorrhizal Fungi Enhances Drought Tolerance in Pedunculate Oak. Int. J. Mol. Sci..

[199] Trudić B., Draškić G., Le Provost G., Stojnić S., Pilipović A., Ivezić A. (2021). Expression profiles of 11 candidate genes involved in drought tolerance of pedunculate oak (*Quercus robur* L.). Possibilities for genetic monitoring of the species. Silvae Genet..

[200] Bose A.K., Scherrer D., Camarero J.J., Ziche D., Babst F., Bigler C., Bolte A., Dorado-Liñán I., Etzold S., Fonti P. (2021). Climate Sensitivity and Drought Seasonality Determine Post-Drought Growth Recovery of *Quercus petraea* and *Quercus robur* in Europe. Sci. Total Environ..

[201] Basu S., Stojanović M., Jevsenak J., Buras A., Pipíšková V., Svetlik J. (2024). Ecological responses of pedunculate oak and narrow-leaved ash to varying groundwater levels in a South Moravian floodplain forest. Book of Abstracts TRACE 2024—Tree Rings in Archaeology, Climatology and Ecology, Proceedings of the Annual Meeting and International Conference of the Association for Tree-Ring Research (ATR), Brașov, Romania, 3–8 June 2024.

[202] Basu S., Stojanović M., Jevšenak J., Buras A., Kulhavý J., Hornová H., Světlík J. (2024). Pedunculate oak is more resistant to drought and extreme events than narrow-leaved ash in Central European floodplain forests. For. Ecol. Manag..

[203] Kesić L., Čater M., Orlović S., Matović B., Stojanović M., Bojović M. (2023). Proximity to riverbed influences physiological response of adult pedunculate oak trees. Topola.

[204] Resente G., Di Fabio A., Scharnweber T., Gillert A., Crivellaro A., Anadon-Rosell A., Trouillier M., Kreyling J., Wilmking M. (2024). The importance of variance and microsite conditions for growth and hydraulic responses following long-term rewetting in pedunculate oak wood. Trees.

[205] Gérard B. (2008). Search for Physiological Markers of Tolerance to Waterlogging in Pedunculate Oak (*Quercus robur* L.) and Sessile Oak (*Quercus petraea* [Mattus.] Liebl. Ph.D. Thesis.

[206] Le Provost G., Lesur I., Lalanne C., Da Silva C., Labadie K., Aury J.M., Leple J.C., Plomion C. (2016). Implication of the suberin pathway in adaptation to waterlogging and hypertrophied lenticels formation in pedunculate oak (*Quercus robur* L.). Tree Physiol..

[207] Horáček P., Šlezingerová J., Gandelová L. (2003). Analysis of cambial activity and formation of wood in *Quercus robur* L. under conditions of a floodplain forest. J. For. Sci..

[208] Perić S., Jazbec A., Medak J., Topić V., Ivanković M. (2006). Analysis of biomass of 16th Pedunculate Oak provenances. Period. Biol..

[209] Popović V., Vemić A., Jovanović S., Lučić A., Rakonjac L., Ivanović B., Miljković D. (2024). The influence of origin on the quality of pedunculate oak (*Quercus robur* L.) seedlings. Reforesta.

[210] Dewan S., De Frenne P., Kepfer-Rojas S., Wasof S., Vander Mijnsbrugge K., Verheyen K. (2021). Weak but Persistent Provenance Effects Modulate the Response of *Quercus robur* (Fagaceae) Seedlings to Elevated Temperature. Ecoevorxiv.

[211] Cuza P. (2023). Differences between provenances and pedunculate oak (*Quercus robur*) trees after the lethal dose (LD50) of electrolyte leakage. Stud. Univ. Mold. Ser. Științe Ale Nat..

[212] Ballian D., Hodžić M.M., Kvesić S. Grouping of Pedunculate Oak Populations (*Quercus robur* L.) Based on Morphological Characteristics and Ecological Vegetation Zoning of Bosnia and Herzegovina. 2017, Abstract. https://www.researchgate.net/publication/327394119_Grouping_of_Pedunculate_Oak_Populations_Quercus_robur_L_Based_on_Morphological_Characteristics_and_Ecological_Vegetation_Zoning_of_Bosnia_and_Herzegovina.

[213] Ballian D., Hodžić M.M. (2022). Preliminary assessment of genetic gain through the selection of different pedunculate oak populations in provenance test. Genet. Appl..

[214] Ugarković D., Tikvić I., Mikac S., Stankić I., Balta D. (2016). The influence of changing climate extremes on the ecological niche of pedunculated oak in Croatia. South-East Eur. For..

[215] Sonesson K., Drobyshev I. (2010). Recent advances on oak decline in southern Sweden. Ecol. Bull..

[216] Repo T., Volanen V., Pulkkinen P. (2022). No difference in the maximum frost hardiness of different pedunculate oak populations in Finland. Silva Fenn..

[217] Franzén M., Hall M., Sunde J., Forsman A. (2024). Regeneration patterns of native and introduced oak species in Sweden: Investigating the roles of latitude, age, and environmental gradients. For. Ecol. Manag..

[218] Firmat C., Delzon S., Louvet J.M., Parmentier J., Kremer A. (2017). Evolutionary dynamics of the leaf phenological cycle in an oak metapopulation along an elevation gradient. J. Evol. Biol..

[219] Caignard T., Kremer A., Firmat C., Nicolas M., Venner S., Delzon S. (2017). Increasing spring temperatures favor oak seed production in temperate areas. Sci. Rep..

[220] Hanley M.E., Cook B.I., Fenner M. (2019). Climate Variation, Reproductive Frequency and Acorn Yield in English Oaks. J. Plant Ecol..

[221] Kelly P.M., Leuschner H.H., Briffa K.R., Harris I.C. (2002). The climatic interpretation of pan-European signature years in oak ring-width series. Holocene.

[222] Büntgen U., Trouet V., Frank D., Leuschner H.H., Friedrichs D., Luterbacher J., Esper J. (2010). Tree-ring indicators of German summer drought over the last millennium. Quat. Sci. Rev..

[223] van der Werf G.W., Sass-Klaassen U., Mohren G.M.J. (2007). The impact of the 2003 summer drought on the intra-annual growth pattern of beech (*Fagus sylvatica* L.) and oak (*Quercus robur* L.) on a dry site in the Netherlands. Dendrochronologia.

[224] Sohar K., Läänelaid A., Eckstein D., Helama S., Jaagus J. (2014). Dendroclimatic signals of pedunculate oak (*Quercus robur* L.) in Estonia. Eur. J. For. Res..

[225] Barsoum N., Eaton E.L., Levanič T., Pargade J., Bonnart X., Morison J.I.L. (2014). Climatic drivers of oak growth over the past one hundred years in mixed and monoculture stands in southern England and northern France. Eur. J. For. Res..

[226] Perkins D., Uhl E., Biber P., Du Toit B., Carraro V., Rötzer T., Pretzsch H. (2018). Impact of Climate Trends and Drought Events on the Growth of Oaks (*Quercus robur* L. and *Quercus petraea* (Matt.) Liebl.) within and beyond Their Natural Range. Forests.

[227] Netsvetov M., Sergeyev M., Nikulina V., Korniyenko V., Prokopuk Y. (2017). The climate to growth relationships of pedunculate oak in steppe. Dendrochronologia.

[228] Matveev S., Milenin A., Timashuk D. (2018). The effects of limiting climatic factors on the increment of native tree species (*Pinus silvestris* L., *Quercus robur* L.) of the Voronezh region. J. For. Sci..

[229] Kalisty A. (2022). Influence of Pluvial and Thermal Conditions on Radial Growth of Pedunculate Oak (*Quercus robur* L.) in Western and North–Eastern Poland. Master’s Thesis.

[230] Ianbaev R.Y., Bakhtina S.Y., Sadykov A.K., Kurbanov E.A. (2023). Climatic response in radial increment of pedunculate oak stands in the Southern Urals. Forest Ecosystems Under Climate Change: Biological Productivity and Remote Monitoring: Compendium of Research Papers.

[231] Rellstab C., Zoller S., Walthert L., Lesur I., Pluess A.R., Graf R., Bodénès C., Sperisen C., Kremer A., Gugerli F. (2016). Signatures of local adaptation in candidate genes of oaks (*Quercus* spp.) with respect to present and future climatic conditions. Mol. Ecol..

[232] Saleh D., Chen J., Leplé J.-C., Leroy T., Truffaut L., Dencausse B., Lalanne C., Labadie K., Lesur I., Bert D. (2022). Genome-wide evolutionary response of European oaks during the Anthropocene. Evol. Lett..

[233] Puchałka R., Prislan P., Klisz M., Koprowski M., Gričar J. (2024). Tree-ring formation dynamics in *Fagus sylvatica* and *Quercus petraea* in a dry and a wet year. Dendrobiology.

[234] Hlásny T., Mátyás C., Seidl R., Kulla L., Merganicova K., Trombik J., Dobor L., Barcza Z., Konopka B. (2014). Climate change increases the drought risk in Central European forests: What are the options for adaptation?. Lesn. Cas. For. J..

[235] Bussotti F., Pollastrini M., Holland V., Brüggemann W. (2015). Functional traits and adaptive capacity of European forests to climate change. Environ. Exp. Bot..

[236] Meger J., Ulaszewski B., Chmura D.J., Burczyk J. (2024). Signatures of local adaptation to current and future climate in phenology-related genes in natural populations of *Quercus robur*. BMC Genom..

[237] Cavender-Bares J., Reich P.B. (2012). Shocks to the system: Community assembly of the oak savanna in a 40-year fire frequency experiment. Ecology.

[238] Cavender-Bares J. (2019). Diversification, Adaptation, and Community Assembly of the American Oaks (Quercus), a Model Clade for Integrating Ecology and Evolution. New Phytol..

[239] Sean C.T. (2011). Genetic vs. phenotypic responses of trees to altitude. Tree Physiol..

[240] Bresson C.C., Vitasse Y., Kremer A., Delzon S. (2011). To what extent is altitudinal variation of functional traits driven by genetic adaptation in European oak and beech?. Tree Physiol..

[241] Chai Y., Zhang X., Yue M., Liu X., Li Q., Shang H., Meng Q., Zhang R. (2015). Leaf traits suggest different ecological strategies for two *Quercus* species along an altitudinal gradient in the Qinling Mountains. J. For. Res..

[242] Kaproth M.A., Fredericksen B.W., Antonio González-Rodríguez A., Hipp A.L., Cavender-Bares J. (2023). Drought response strategies are coupled with leaf habit in 35 evergreen and deciduous oak (*Quercus*) species across a climatic gradient in the Americas. New Phytol..

[243] Liu D., Guo H., Yan L.-P., Gao L., Zhai S., Xu Y. (2024). Physiological, Photosynthetic and Stomatal Ultrastructural Responses of *Quercus acutissima* Seedlings to Drought Stress and Rewatering. Forests.

[244] Xiong S., Wang Y., Chen Y., Gao M., Zhao Y., Wu L. (2022). Effects of Drought Stress and Rehydration on Physiological and Biochemical Properties of Four Oak Species in China. Plants.

[245] Tikhomirova T.S., Krutovsky K.V., Shestibratov K.A. (2023). Molecular Traits for Adaptation to Drought and Salt Stress in Birch, Oak and Poplar Species. Forests.

[246] Klekowski E.J. (1988). Genetic load and its causes in long-lived plants. Trees.

[247] Burian A., de Reuille P.B., Kuhlemeier C. (2016). Patterns of Stem Cell Divisions Contribute to Plant Longevity. Curr. Biol..

[248] Padovan A., Keszei A., Foley W.J., Kulheim C. (2013). Differences in gene expression within a striking phenotypic mosaic *Eucalyptus* tree that varies in susceptibility to herbivory. BMC Plant Biol..

[249] Tobias P.A., Guest D.I. (2014). Tree immunity: Growing old without antibodies. Trends Plant Sci..

[250] Schmid-Siegert E., Sarkar N., Iseli C., Calderon S., Gouhier-Darimont C., Chrast J., Cattaneo P., Schütz F., Farinelli L., Pagni M. (2017). Low number of fixed somatic mutations in a long-lived oak tree. Nat. Plants.

[251] Biondi F., Meko D.M., Piovesan G. (2023). Maximum Tree Lifespans Derived from Public-Domain Dendrochronological Data. iScience.

[252] Popov V.N., Syromyatnikov M.Y., Franceschi ‪C., Moskalev A.A., Krutovsky K.V. (2022). Genetic mechanisms of aging in plants: What can we learn from them?. Ageing Res. Rev..

[253] Batalova A.Y., Krutovsky K.V. (2023). Genetic and epigenetic mechanisms of longevity in forest trees. Int. J. Mol. Sci..

[254] Cui J., Li X., Lu Z., Jin B. (2024). Plant secondary metabolites involved in the stress tolerance of long-lived trees. Tree Physiol..

[255] Liu S., Xu H., Wang G., Jin B., Cao F., Wang L. (2025). Tree Longevity: Multifaceted Genetic Strategies and Beyond. Plant Cell Environ..

[256] Volkava D., Riha K. (2024). Growing old while staying young: The unique mechanisms that defy aging in plants. EMBO Rep..

[257] Ianbaev R.Y., Bakhtina S.Y., Sadykov A.K., Yanbaev Y.A. (2022). Analysis of the relationship between climatic factors and genetic diversity of pedunculate oak populations in different parts of the Republic of Bashkortostan. Bull. Perm Univ. Biol..

[258] Kajba D., Katičić I., Bogdan S. (2011). Estimation of Genetic Parameters in Open Pollinated Progeny Trials from Plus trees of Pedunculate Oak (*Quercus robur* L.) Selected in Posavina and Podravina and Podunavlje Seed Zones. Croat. J. For. Eng..

[259] Fedorkov A.L. (2019). Phenotypical selection in forest breeding. Lesovedenie.

[260] Herzog S. (1996). Genetic inventory of European oak populations: Consequences for breeding and gene conservation. Ann. For. Sci..

[261] Kostrikin V.A., Shirnin V.K., Kryukova S.A. (2021). Criteria for Assessment of Plus Oak Stands. Lesn. Zhurnal (For. J.).

[262] Shirnin V.C., Kryukova S.A. (2015). Modeling the perfect grade common oak high quality wood. Current Research Trends of the XXI Century: Theory and Practice.

[263] Tsarev A.P., Laur N.V., Tsarev V.A., Tsareva R.P. (2021). The Current State of Forest Breeding in the Russian Federation: The Trend of Recent Decades. Lesn. Zhurnal (For. J.).

[264] Sukhorukikh Y.I., Biganova S.G. (2023). Selection Criteria for Plus Stands in Field-Protective Forest Belts in the North-Western Caucasus. Lesotekhnicheskii Zhurnal (For. Eng. J.).

[265] Trudić B., Avramidou E., Fussi B., Neophytou C., Stojnić S., Pilipović A. (2021). Conservation of *Quercus robur* L. genetic resources in its south-eastern refugium using SSR marker system–a case study from Vojvodina province, Serbia. Austrian J. For. Sci..

[266] Stojnić S., Trudić B., Galović V., Šimunovački Đ., Đorđević B., Rađević V., Orlović S. (2014). Conservation of pedunculate oak (*Quercus robur* L.): Genetic resources at the territory of public enterprise ‘Vojvodinašume’. Topola (Poplar).

[267] Fedorkov A.L. (2024). Forest Tree Breeding and Genetic Diversity of Wood Species. Lesn. Zhurnal (For. J.).

[268] Lebedev V.G., Lebedeva T.N., Chernodubov A.I., Shestibratov K.A. (2020). Genomic Selection for Forest Tree Improvement: Methods, Achievements and Perspectives. Forests.

[269] Grattapaglia D. (2022). Twelve Years into Genomic Selection in Forest Trees: Climbing the Slope of Enlightenment of Marker Assisted Tree Breeding. Forests.

[270] Sharma U., Sankhyan H.P., Kumari A., Thakur S., Thakur L., Mehta D., Sharma S., Sharma S., Sankhyan N. (2024). Genomic selection: A revolutionary approach for forest tree improvement in the wake of climate change. Euphytica.

[271] Alexandre H., Truffaut L., Klein E., Ducousso A., Chancerel E., Lesur I., Dencausse B., Louvet J.-M., Nepveu G., Torres-Ruiz J.M. (2020). How does contemporary selection shape oak phenotypes?. Evol. Appl..

[272] Caignard T., Truffaut L., Delzon S., Dencausse B., Lecacheux L., Torres-Ruiz J.M., Kremer A. (2024). Fluctuating selection and rapid evolution of oaks during recent climatic transitions. Plants People Planet.

